# Supplementary material for: Hybrid Automata Library: A flexible platform for hybrid modeling with real-time visualization
Source: PLoS Comput Biol. 2020 Mar 10;16(3):e1007635. doi: 10.1371/journal.pcbi.1007635 (PMC7105119; doi:10.1371/journal.pcbi.1007635)
Supplement: S1 Text — (PDF) [file pcbi.1007635.s001.pdf]

Supplementary Materials for:

Bravo et al., Hybrid Automata Library: A modular platform for efficient hybrid modeling with real-time visualization, PLoS Comput. Biol. (2019)

February 26, 2020

Rafael Bravo, Etienne Baratchart, Jeffrey West, Ryan O. Schenck, Anna K. Miller, Jill Gallaher, Chandler D. Gatenbee, David Basanta, Mark Robertson-Tessi, Alexander R. A. Anderson

Model Performance Scaling

To demonstrate HAL's performance scaling as model size increases, we used a slightly modified version of the Competitive Release model example from the main text. In this version, the model is initiated with a completely filled lattice of resistant cells, and drug application is constant (DRUG_DURATION is set equal to DRUG_PERIOD). Under these conditions, we look at how two functions; a DiffusionStep function and a StepAllCells function; scale in performance as the domain size is increased. The implementation of these functions is shown below. See the main text for explanations of the functions and variables being used.

```
    public void DiffusionStep(int tick){
        if (tick > DRUG_START && (tick - DRUG_START) % DRUG_PERIOD <
            DRUG_DURATION) {
            drug.DiffusionADI(DRUG_DIFF_RATE, DRUG_BOUNDARY_VAL);
        } else {
            drug.DiffusionADI(DRUG_DIFF_RATE);
        }
        drug.Update();
    }
    public void StepAllCells(int tick){
        ShuffleAgents(rn);
        for (ExampleCell cell : this) {
            cell.CellStep();
        }
    }
```

These functions were profiled for 100,000 time-steps using the VisualVM sampler to calculate the average of 3 runs at domain sizes 60×60, 90×90, 120×120, 150×150, and 180×180. The domains model areas of 1.44 mm^2, 3.24 mm^2, 9 mm^2, and 12.96 mm^2 of cells with a radius of 10 µm. At the largest domain size, the model takes around 2.4 ms to run per timestep, around 25000 timesteps per minute. If we assume that each timestep represents 2 hours, as in the main paper model, then we can simulate roughly 5.8 years of real time in 1 minute. These results are plotted in Fig B and show an approximately linear O(n) scaling for diffusion and cell step calculations.

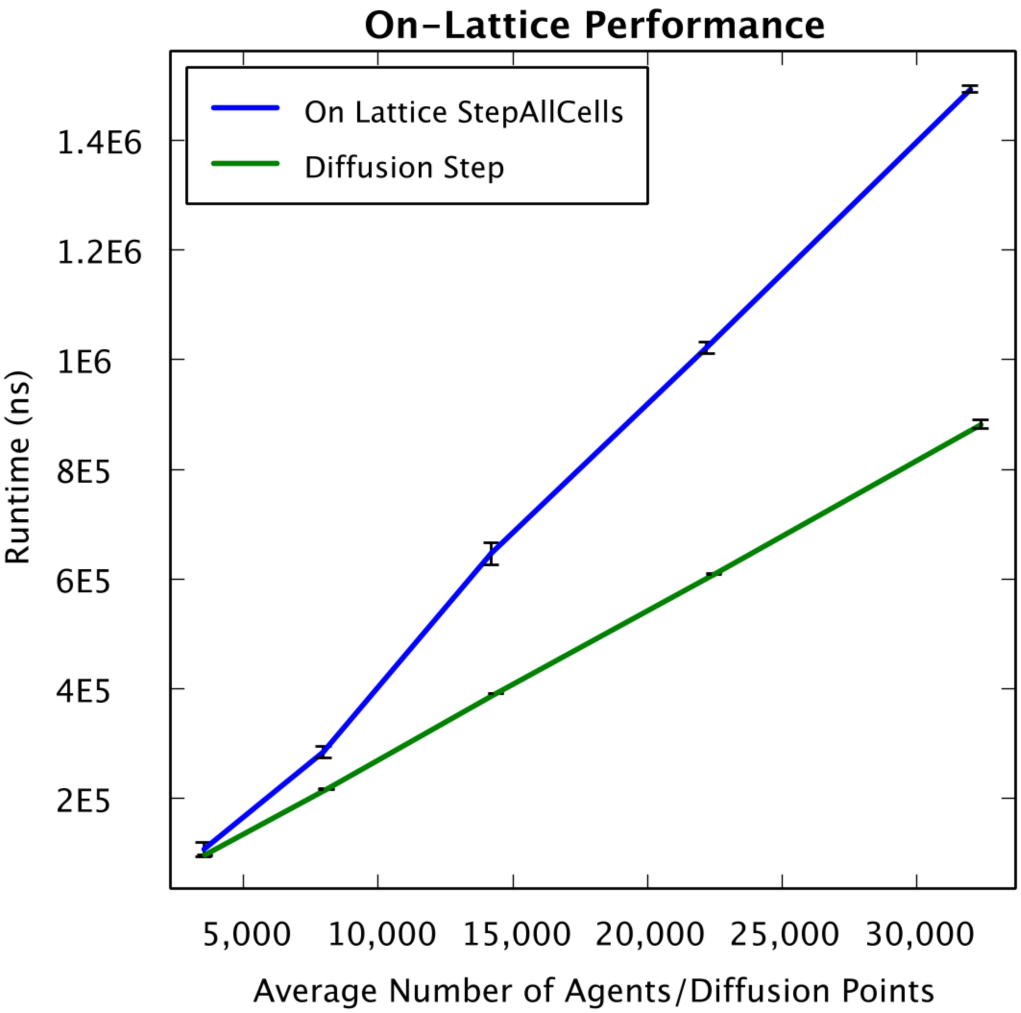

Figure A. The runtime in nanoseconds for 100,000 timesteps of the DiffusionStep and StepAllCells functions on a 2017 MacBook Pro (Version 10.14.4, 2.9GHz Intel i7, 16GB 2133 MHz LPDDR3) are shown for different numbers of lattice positions. The Area on the x axis refers to the approximate number of cells and the number of PDE lattice positions to be updated at every time-step. The means and 95% confidence intervals are plotted from 3 runs at each domain size.

We also profiled the same model sizes for 100,000 time-steps using a modified off-lattice spherical agent version of the same model at domain sizes 60×60, 90×90, 120×120, 150×150, and 180×180. The domain model areas represent 1.44 mm^2, 3.24 mm^2, 9 mm^2, and 12.96 mm^2 for cells with a radius of 10μm. At the largest domain size, the model takes around 12.7 ms to run per timestep, around 4700 timesteps per minute. If we assume that each timestep represents 2 hours as in the main paper model, then we can model roughly 1 year in 1 minute. This model uses spherical agents that push off each other using a Hooke's law force calculation function. Cells will only divide in this version if the pressure on the cells is below a threshold as imposed as part of the cell division if-statement. Both models can be found in the Testing/PerformanceTestModels folder of the HAL codebase.

```
    public void StepAllCells(int tick){
        for (ExampleCell cell : this) {
            cell.BirthDeath();
        }
        for (ExampleCell cell : this) {
            cell.Movement();
        }
    }

class ExampleCell extends SphericalAgent2D<ExampleCell, ExampleModel> {
    public int type;

    public void BirthDeath() {
        //Consumption of Drug
        G.drug.Mul(Xpt(),Ypt(), G.DRUG_UPTAKE);
        //Chance of Death, depends on resistance and drug concentration
        if (G.rn.Double() < G.DEATH_PROB + (type == RESISTANT ? 0 : G.drug.Get(Xpt(),Ypt()) * G.DRUG_DEATH)) {
            Dispose();
            return;
        }
        double pressure=SumForces(1,(overlap, other) -> overlap*G.FORCE_SCALER);
        //contact inhibition and division probability influence division event
        if (G.rn.Double()*pressure*1000 < (type == RESISTANT ? G.DIV_PROB_RES : G.DIV_PROB_SEN)) {
           ExampleCell c=Divide(radius*1.0/3,G.scratch,G.rn);
           c.radius=radius;
           c.xVel=0;
           c.yVel=0;
        }

    }
    public void Movement() {
        ForceMove();
        ApplyFriction(0);
    }
}
```

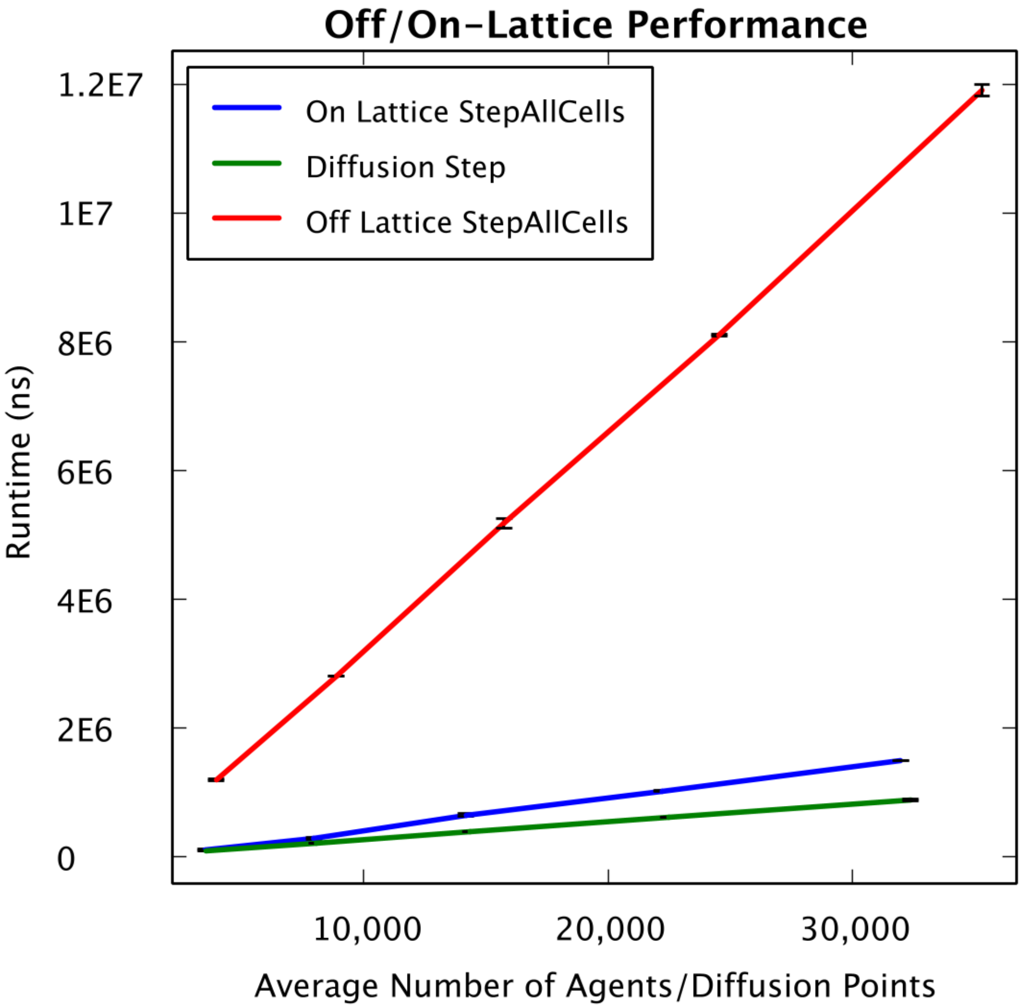

Figure B. The average runtime in nanoseconds per timestep from 100,000 timesteps of the StepAllCells functions on a 2017 MacBook Pro (Version 10.14.4, 2.9GHz Intel i7, 16GB 2133 MHz LPDDR3) are shown for different average population sizes. The means and 95% confidence intervals are plotted from 3 runs at each population size.

To test how HAL's performance scales to very large domains, we present the same data at log scale with average timestep durations from two additional model runs with 1 million agents and diffusible positions running for 1000 timesteps and 10 million agents running for 100 timesteps. These models represent areas of 400 mm^2 and 40000 mm^2 for cells with a radius of 10μm). At the largest domain size, the on lattice version and off lattice version take around 3.5 and 18 seconds to run one timestep (representing 2 hours) respectively. At 100 million agents, the laptop memory was insufficient to store all of the agents causing the model run to terminate. For this reason we recommend running models with more agents on specialized hardware with expanded RAM if available. HAL's design is currently tailored toward single threaded modeling. Multi-threading becomes necessary for fast performance at the scale of millions of agents. We may expand the multi-threading capabilities of HAL in the future to support high performance at this scale.

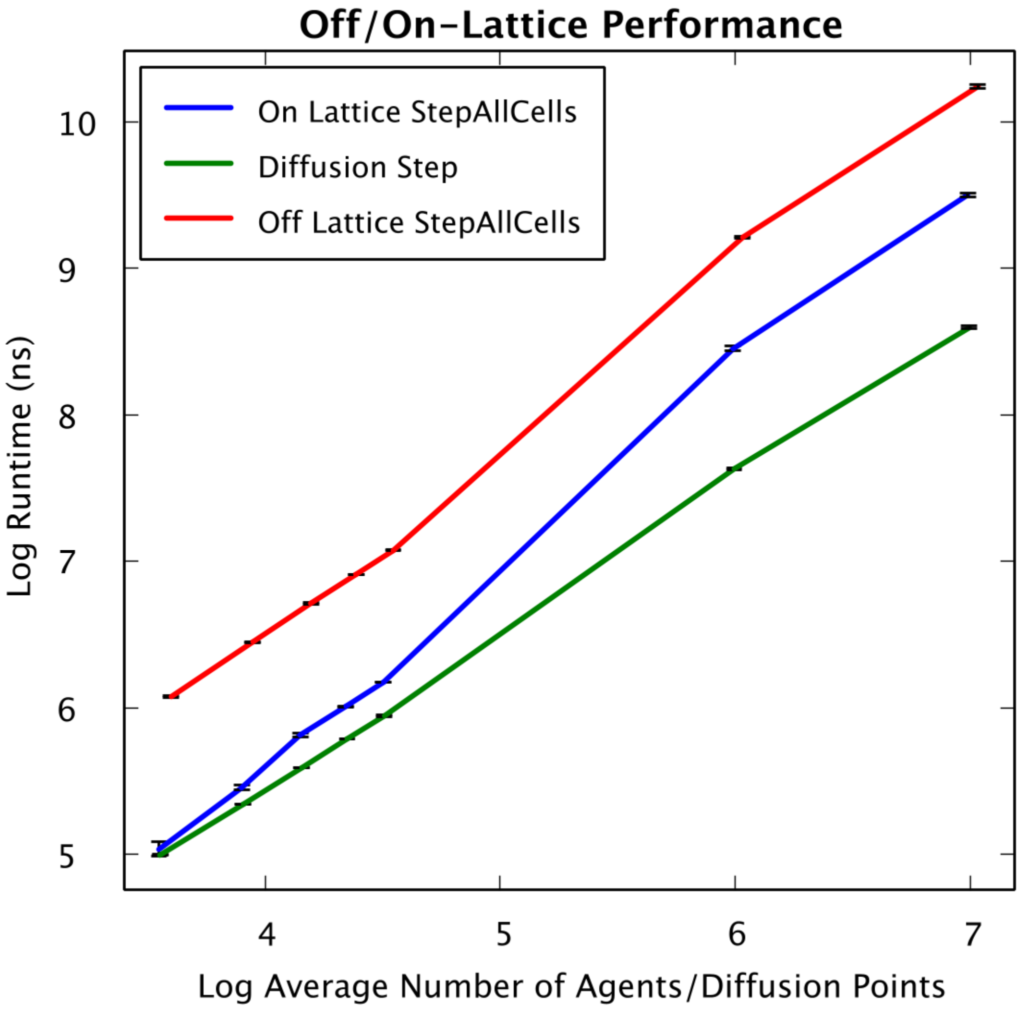

Figure C. The log(base 10) average runtime in nanoseconds per timestep from 1,000 timesteps of the StepAllCells functions with a million agents and diffusion points on a 2017 MacBook Pro (Version 10.14.4, 2.9GHz Intel i7, 16GB 2133 MHz LPDDR3) are shown for different average population sizes. The means and 95% confidence intervals are plotted from 3 runs at each population size. Previous performance test results are also shown.

Type Hierarchy

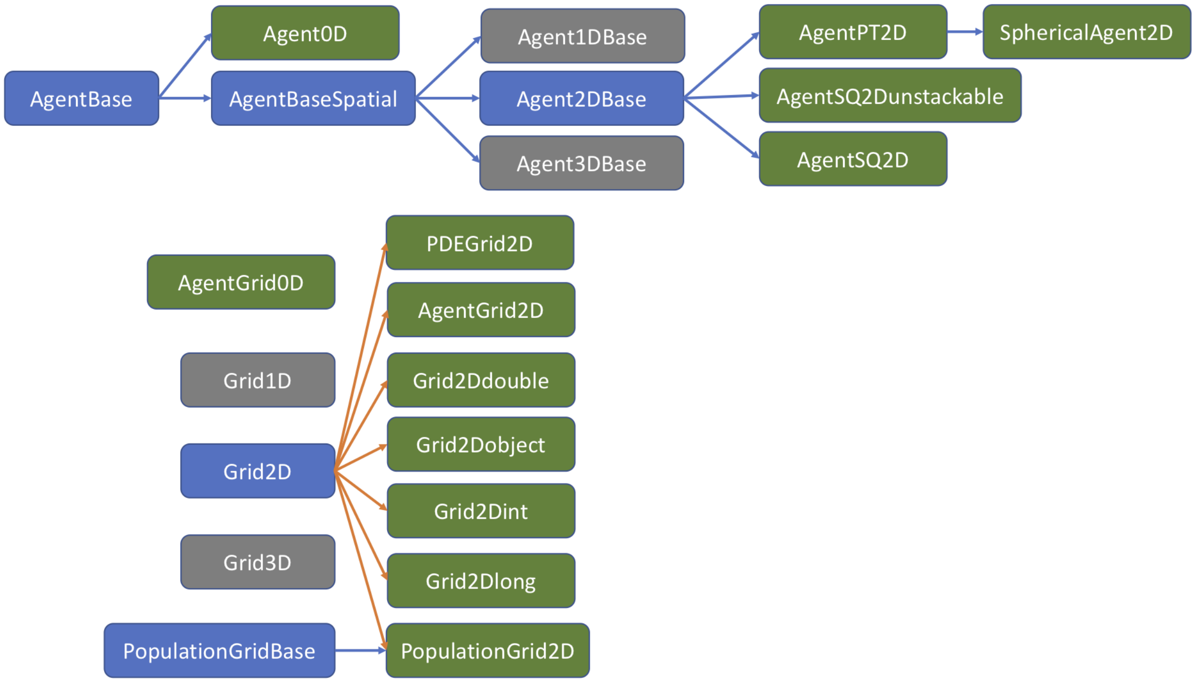

Figure D. A visual representation of the type extension hierarchy behind the hybrid modeling Agent and Grid types currently available in HAL. Blue arrows show an extension relationship, with extended classes able to utilize all the methods and variables of the types that they extend. Orange arrows show an interface implementation relationship, meaning that all classes implement methods defined by the base interface, and have any default functions defined in the interface. Blue boxes are abstract types or interfaces, which cannot be directly instantiated. Green boxes are concrete types that can be instantiated and are directly used in model building. Grey boxes are abstract types whose concrete type extension classes are not shown, but are similar to the 2D concrete type extensions shown.

Agent Implementation Differences

Here we will discuss the differences in implementation between the base agent types. The functional differences between the agent types are entirely defined in Table 2 of the main text. There are some small differences in implementation between types, primarily to make small performance gains when the type restrictions allow for this. PT agents use floating point numbers (Java doubles) to represent their position, since they need to move continuously. SQ agents use integers to represent their position for a small performance benefit (integer computation is faster than double computation on most computer architectures). The "stackable" SQ and PT agents have built-in links to allow multiple agents to occupy the same position; agents in the same position link to each other in a linked list, with agents serving as the links. Unstackable SQ agents are exactly like the stackable SQ agents in implementation, but do not contain these links or maintain them when they change lattice position, which saves on computation time and their size in memory. When choosing which type of agent to use, it is important to keep in mind that the different types exist mostly because they restrict the spatial

representation of agents in ways that are commonly useful, rather than for performance benefits, so we recommend choosing agent types based on the spatial constraints you want to impose in your model design.

Working Across Agent Types

The extension hierarchy allows for tools to be developed that target multiple agent types by working with the base types that all of the extended types are derived from. Another way for cross-type compatibility is through the use of interfaces that ensure that any agents that work with a given tool can provide the information that the tool needs to function. Both of these design patterns can be found in the source code, for example the AgentGrid1D/2D/3D code works with the abstract AgentBaseSpatial agent type, rather than any of the more specific agent types mentioned, allowing for all of the spatial agent types to be compatible. As an example of the use of interfaces, the VarSetManager class, which allows modular adding of additional state variables to agents, requires agents to implement the VarSet interface so that the variables can be assigned to each agent by the VarSetManager. The VarSetManager can be found in Framework/Tools/Modularity.

Interactions in HAL between different agent types are typically facilitated using spatial queries across separate grids that house these different agent types, so arbitrary interactions are possible from a decoupled perspective. However a single AgentGrid cannot house multiple agent types directly. The shared API across agent types should make migrating between them relatively painless, as most built-in agent functions are shared across types.

AgentGrid/PDEGrid Implementation Differences

The AgentGrids/PDEGrids use the same underlying structure; each uses an array to maintain spatial arrangements, with different access functions for different numbers of dimensions. HAL does not currently provide built-in support for arbitrary spatial graph structures, supporting only 1D 2D and 3D square lattices for Diffusion and ABMs. It is worth noting, however that the neighborhood generation utility functions include triangular and hexagonal neighborhoods, since these can be mapped to a square lattice with different connections linking positions. Using these neighborhoods a modeler can increase the number of graph connections between agents. In the future it would be sensible to extend the AgentGrid1D to facilitate arbitrary graphs, as the AgentGrid1D has the most straightforward access functions to map an arbitrary graph on top of.

PDE Convergence

In order to assess the right implementation of the different PDE schemes, we calculated orders of convergence in space and time for the numerical schemes implemented in HAL. In order to compute orders of convergence in space, we compared successive numerical solutions with increasing grid sizes, where the difference between two solutions was determined regarding a certain norm. In order to compute orders of convergence in time, we compared successive numerical solutions with increasing the number of time steps, for a fixed grid size, where the difference between two solutions was determined regarding a certain norm. We considered the norms $L1$, $L2$ and L_∞ to define convergence here. In one, two and three dimensions, we performed test cases for diffusion (Dirichlet, Neumann and periodic boundary conditions) and for advection (Dirichlet, and periodic boundary conditions). For the sake of simplicity we established the following convention: we cartesian coordinates defined on $[0, 1]$ along each dimension. For the spatial convergence the successive grid sizes were set as follows for 1D:

- 100 points
- 300 points
- 900 points
- 2700 points
- 8100 points

Similarly for 2D:

- 10×10 points
- 30×30 points
- 90×90 points
- 270×270 points

Similarly for 3D:

- $10 \times 10\times$ 10 points
- $30 \times 30 \times 30$ points
- $90 \times 90 \times 90$ points
- $270 \times 270 \times 270$ points

For all the time convergence tests, the grid size was fixed at 10 points per dimension. Initial adimensionalized constants D_a and v_a were set to 0.1 for diffusion and advection respectively. The number of time steps was set to 100 initially and was iteratively multiplied by a factor of 2.

We then plotted the successive errors between grid sizes as a function of the grid size, for $L1$, $L2$ and L_∞ norms, all in log scale. The slope of the straight line corresponds to the order of convergence of the implemented scheme. For spatial convergence, When the grids were refined, the time step size was adapted accordingly (for FTCS and Upwind schemes only, not for Crank-Nicolson and ADI), in order to ensure the stability of the scheme. For FTCS diffusion scheme, this stability condition is of the form:

$$\frac{D\Delta t}{\Delta x^2} < c, \tag{1}$$

where $c = 0.5$ in 1D and $c = 0.25$ in 2D, and $c = 0.166$ in 3D. For Upwind advection scheme, the time step size was set accordingly, in order to ensure the CFL stability condition:

$$\frac{v\Delta t}{\Delta x} < c, \tag{2}$$

where $c = 1$ in 1D, $c = 0.5$ in 2D, and $c = 0.33$ in 3D, and $v = max(v_3, v_y, v_z)$

Diffusion

As theoretically expected, for all dimensions and boundary conditions types, FTCS, Crank-Nicolson and ADI schemes converged at the order 2 in space for all tests. As expected as well, FTCS scheme converged at the order 1 in time, whereas Crank-Nicolson and ADI schemes converged at the order 2 in time.

1D

In this set of test cases, we are solving the following equation:

$$\frac{\partial C}{\partial t} - D\frac{\partial C}{\partial x^2} = 0 \tag{3}$$

We tested the forward-time centered space scheme (FTCS), for which the normalized adimensionalized diffusion constant $D_a = \frac{D\Delta t}{\Delta x^2}$ was set to be equal to 0.1 during spatial convergence, and then Crank-Nicolson scheme, that is unconditionally stable in L_2 norm, for which we assessed the order of convergence in space without adapting the time step.

Dirichlet boundary conditions 1-0 In this test case, the initial density is 0 everywhere on the domain. Dirichlet conditions are imposed at the boundary as follows:

- C(0,t)=1
- C(1,t)=0

The following figures show the error plots in log scale:

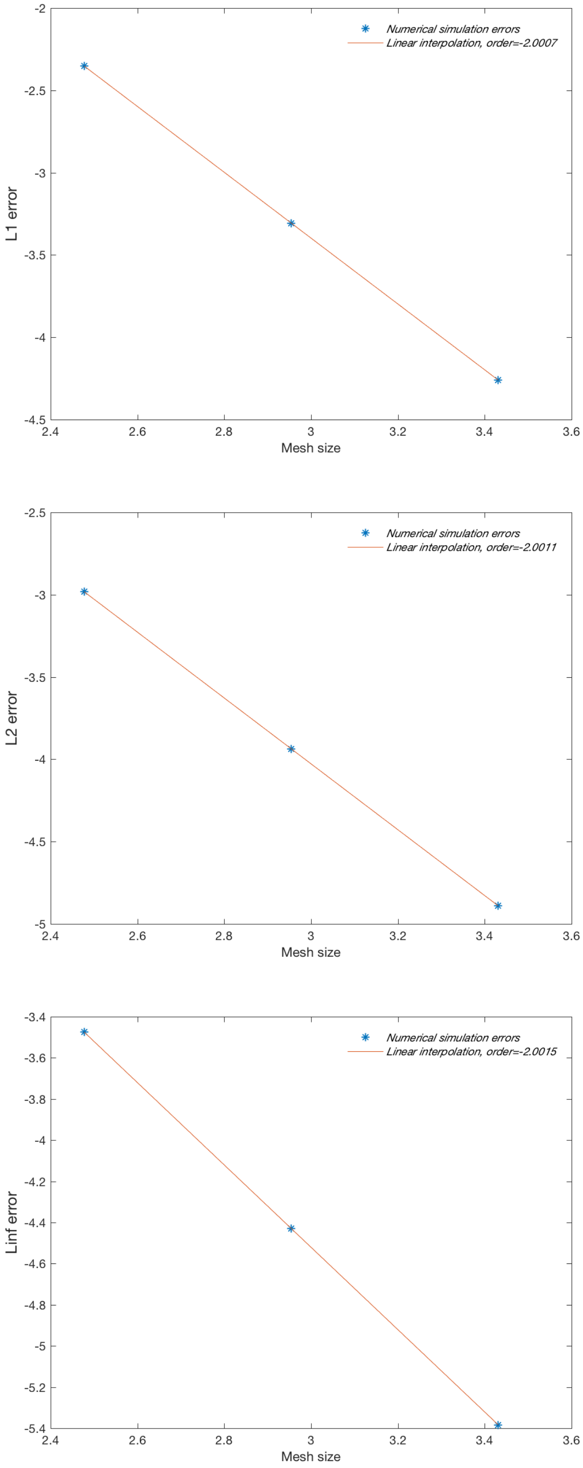

Figure E. Spatial convergence: L_1, L_2, and L_∞ errors of convergence plotted against the grid size in $\log_{10}$ scale obtained with the FTCS scheme for 1D diffusion for Dirichlet boundary conditions (1 at left, 0 at right). $D_a = 0.1$. Initial (for the coarsest grid) time step number: 500. Grid sizes: 100, 300 and 900,2700 points

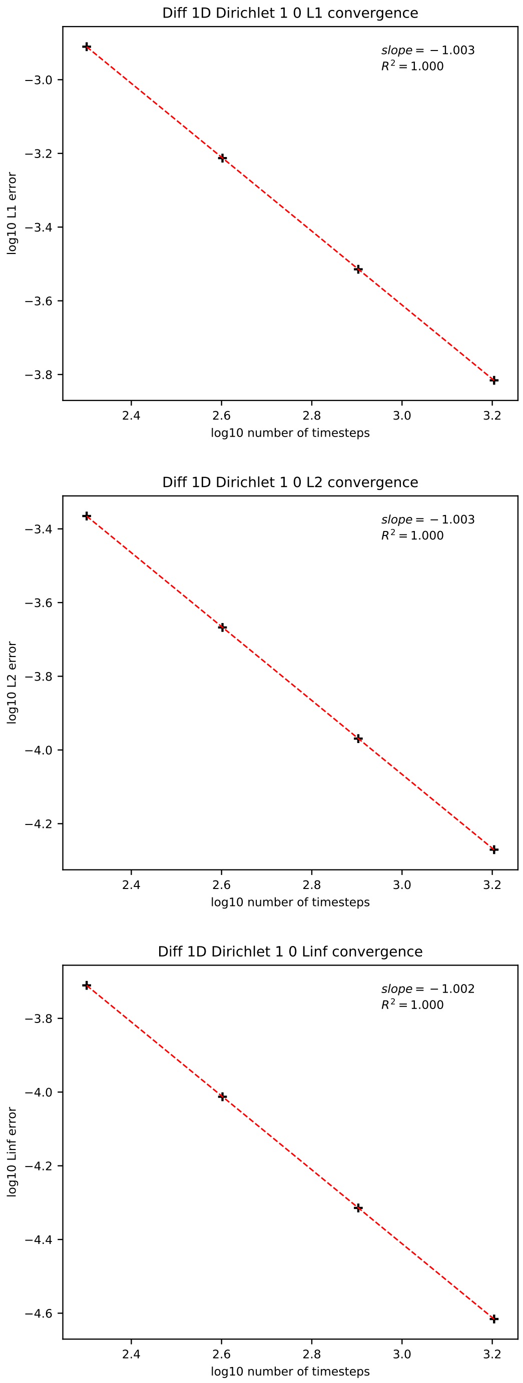

Figure F. Time convergence: L_1, L_2, and L_∞ errors of convergence plotted against the number of time steps in $\log_{10}$ scale obtained with the FTCS scheme for 1D diffusion for Dirichlet boundary conditions (1 at left, 0 at right). $D_a = 0.1$. Initial time step number: 100. Fixed grid size: 10 points

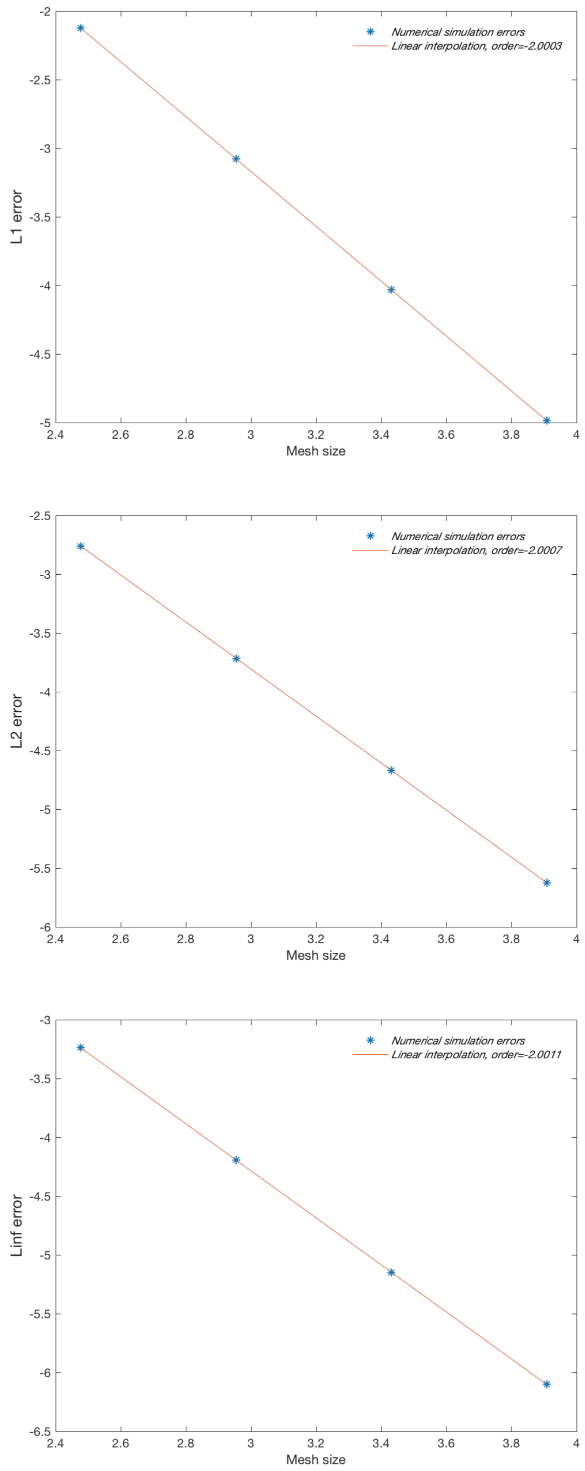

Figure G. Spatial convergence: L_1, L_2, and L_∞ errors of convergence plotted against the grid size in $\log_{10}$ scale obtained with the Crank-Nicolson scheme for 1D diffusion for Dirichlet boundary conditions (1 at left, 0 at right). Initial $D_a = 0.1$. Fixed time step number: 500. Grid sizes: 100, 300 and 900,2700 points

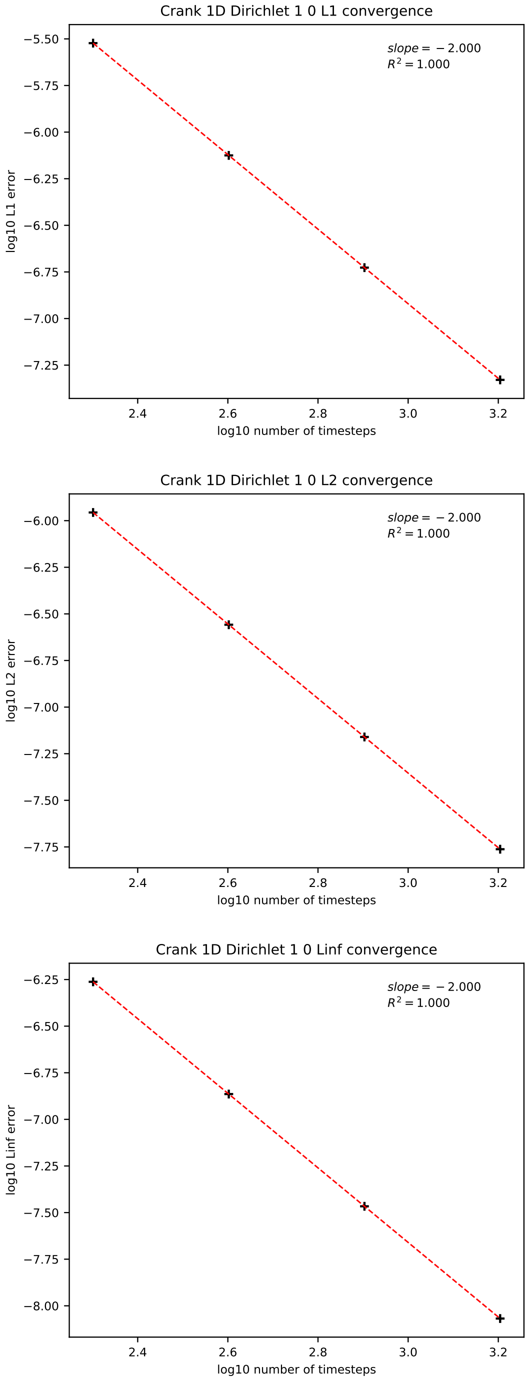

Figure H. Time convergence: L_1, L_2, and L_∞ errors of convergence plotted against the number of time steps in $\log_{10}$ scale obtained with the Crank-Nicolson scheme for 1D diffusion for Dirichlet boundary conditions (1 at left, 0 at right). $D_a = 0.1$. Initial time step number: 100. Fixed grid size: 10 points

Dirichlet boundary conditions 0-0 In this test case, the initial density follows a

Normal distribution centered on the middle of the domain:

$$C(0, x) = \exp\left(\frac{-(x-0.5)^2}{1000}\right) \tag{4}$$

We imposed the value of the field to be 0 at the boundaries.

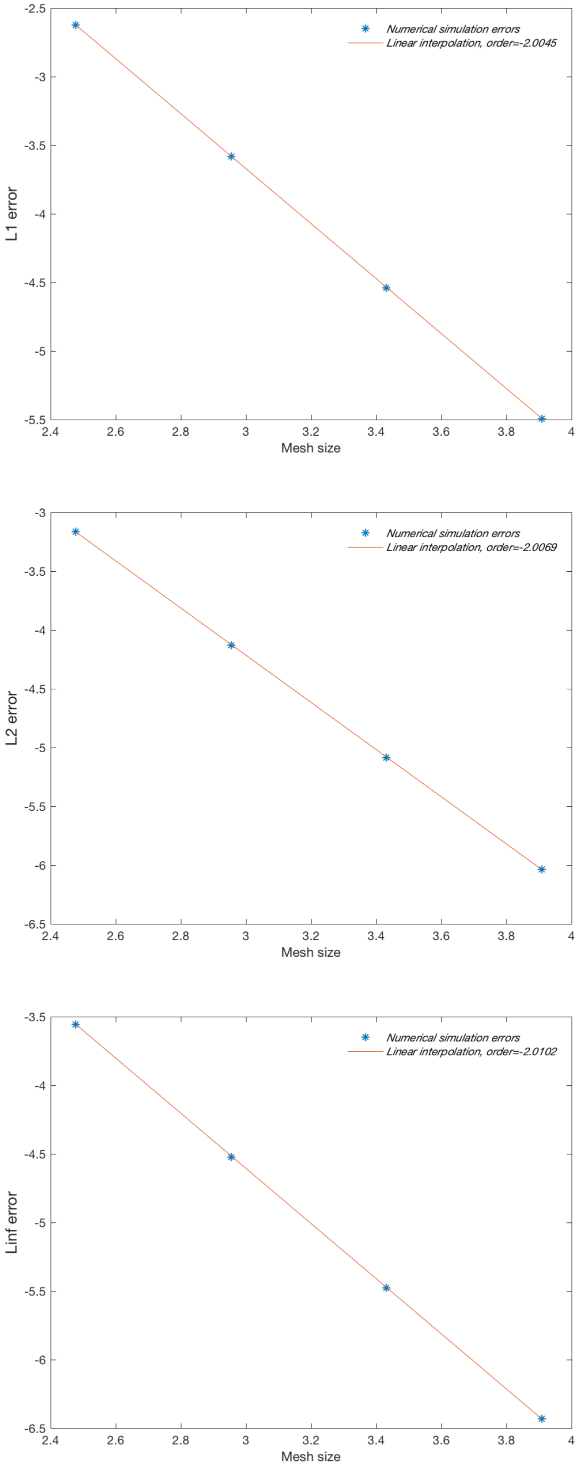

Figure I. Spatial convergence: L_1, L_2, and L_∞ errors of convergence plotted against the grid size in $\log_{10}$ scale obtained with the FTCS scheme for 1D diffusion for Dirichlet boundary conditions (0 at both sides). $D_a = 0.1$. Initial (for the coarsest grid) time step number: 50. Grid sizes: 100, 300, 900, 2700 and 8100 points

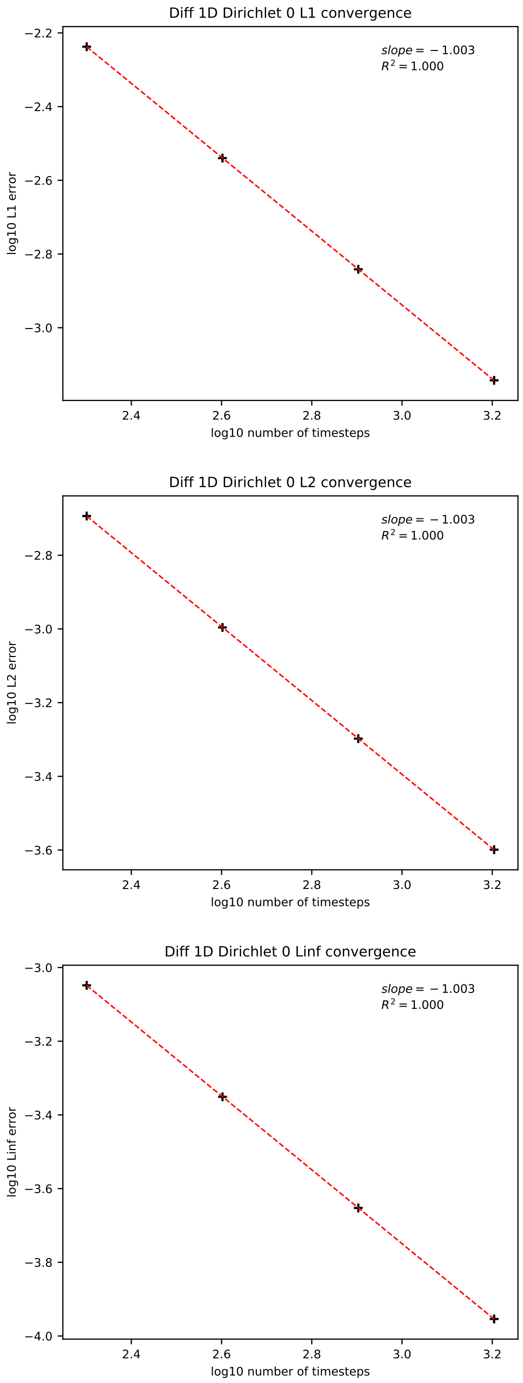

Figure J. Time convergence: L_1, L_2, and L_∞ errors of convergence plotted against the number of time steps in $\log_{10}$ scale obtained with the FTCS scheme for 1D diffusion for Dirichlet boundary conditions (0 at both sides). $D_a = 0.1$. Initial time step number: 100. Fixed grid size: 10 points

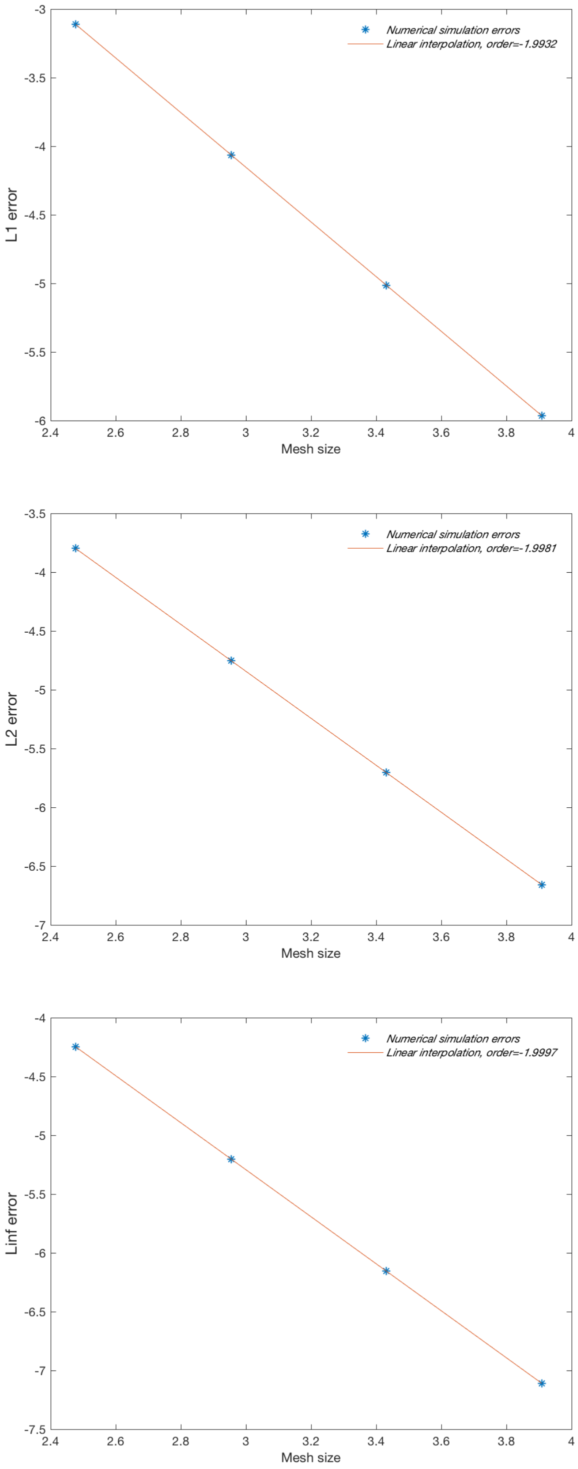

Figure K. Spatial convergence: L_1, L_2, and L_∞ errors of convergence plotted against the grid size in $\log_{10}$ scale obtained with the Crank-Nicolson scheme for 1D diffusion for Dirichlet boundary conditions (0 at both sides). Initial $D_a = 0.1$ Fixed time step number: 50. Grid sizes: 100, 300, 900, 2700 and 8100 points

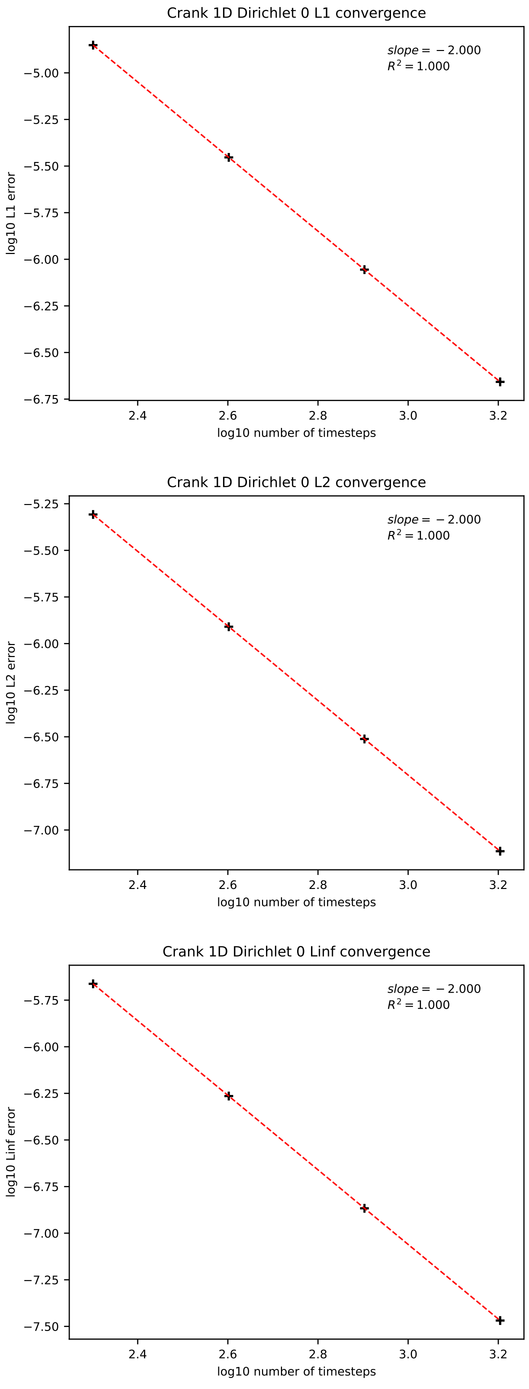

Figure L. Time convergence: L_1, L_2, and L_∞ errors of convergence plotted against number of time steps in $\log_{10}$ scale obtained with the Crank-Nicolson scheme for 1D diffusion for Dirichlet boundary conditions (0 at both sides). $D_a = 0.1$. Initial time step number: 100. Fixed grid size: 10 points

Neumann boundary conditions In this test case, the initial density follows a

Normal distribution centered on the middle of the domain:

$$C(0, x) = \exp\left(\frac{-(x - 0.5)^2}{1000}\right) \tag{5}$$

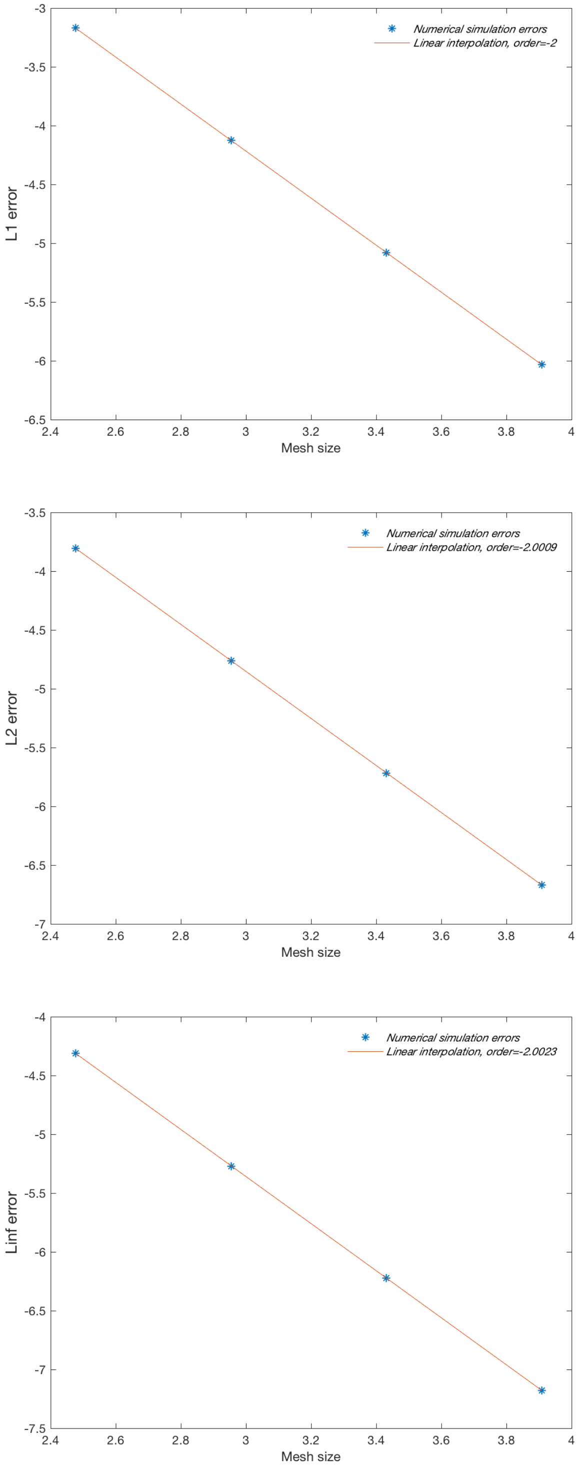

Figure M. Spatial convergence: L_1, L_2, and L_∞ errors of convergence plotted against the grid size in $\log_{10}$ scale obtained with the FTCS scheme for 1D diffusion for Neumann boundary conditions (0 flux at both sides). $D_a = 0.1$. Initial (for the coarsest grid) time step number: 50. Grid sizes: 100, 300, 900, 2700 and 8100 points

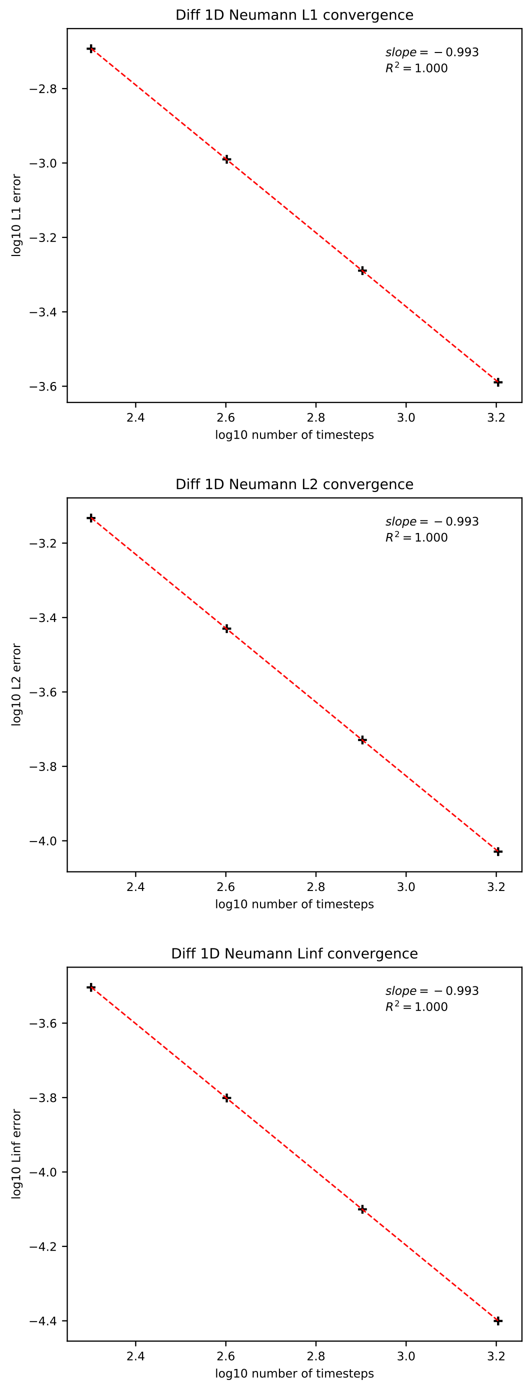

Figure N. Time convergence: L_1, L_2, and L_∞ errors of convergence plotted against the number of time steps in $\log_{10}$ scale obtained with the FTCS scheme for 1D diffusion for Neumann boundary conditions (0 flux at both sides). $D_a = 0.1$. Initial time step number: 100. Fixed grid size: 10 points

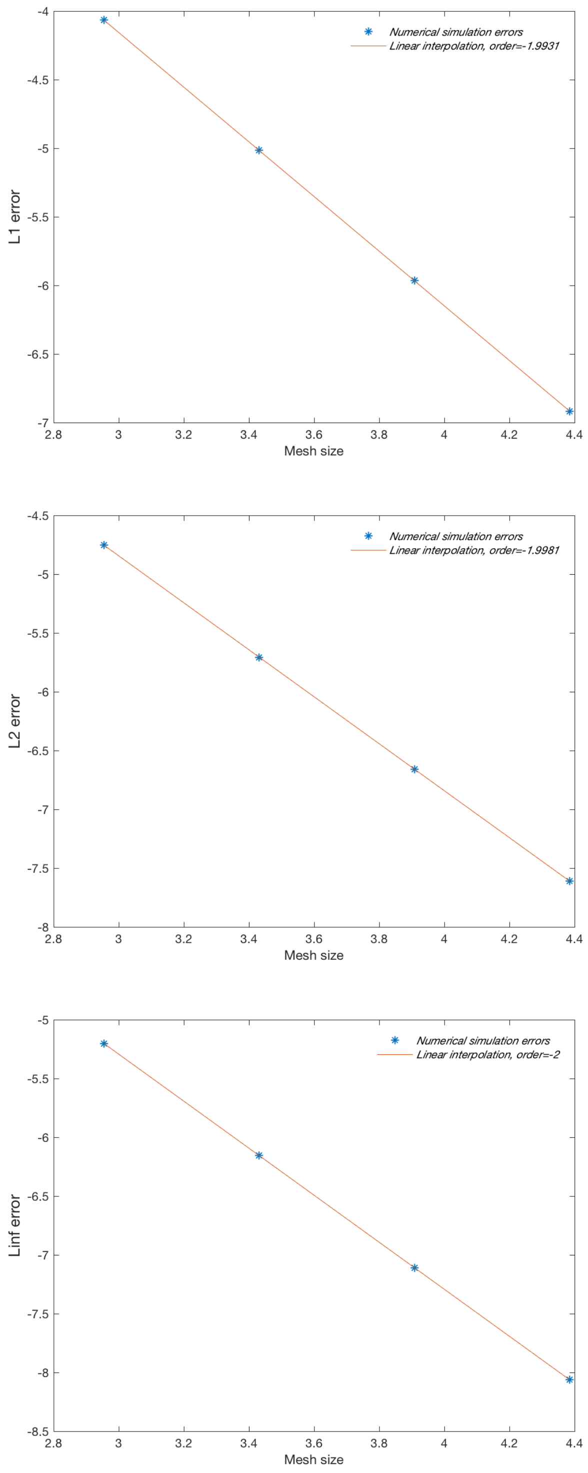

Figure O. Spatial convergence: L_1, L_2, and L_∞ errors of convergence plotted against the grid size in $\log_{10}$ scale obtained with the Crank-Nicolson scheme for 1D diffusion for Neumann boundary conditions (0 flux at both sides). Initial $D_a = 0.1$ Fixed time step number: 50. Grid sizes: 100, 300, 900, 2700 and 8100 points

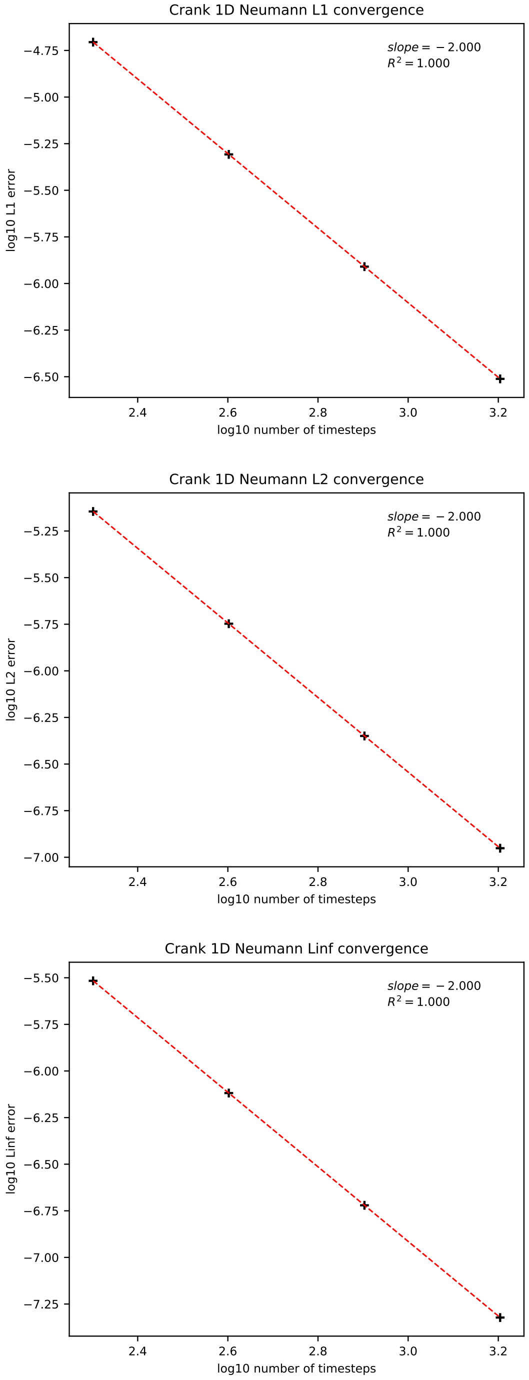

Figure P. Time convergence: L_1, L_2, and L_∞ errors of convergence plotted against the number of time steps in $\log_{10}$ scale obtained with the Crank-Nicolson scheme for 1D diffusion for Neumann boundary conditions (0 flux at both sides). $D_a = 0.1$. Initial time step number: 100. Fixed grid size: 10 points

Periodic boundary conditions In this test case, we consider the regular

symmetrical initial condition defined as follows:

$$C(0,x) = \exp\left(-\frac{(x-0.5)^2}{1000}\right) \tag{6}$$

and simulate the diffusion equation with periodic boundary conditions.

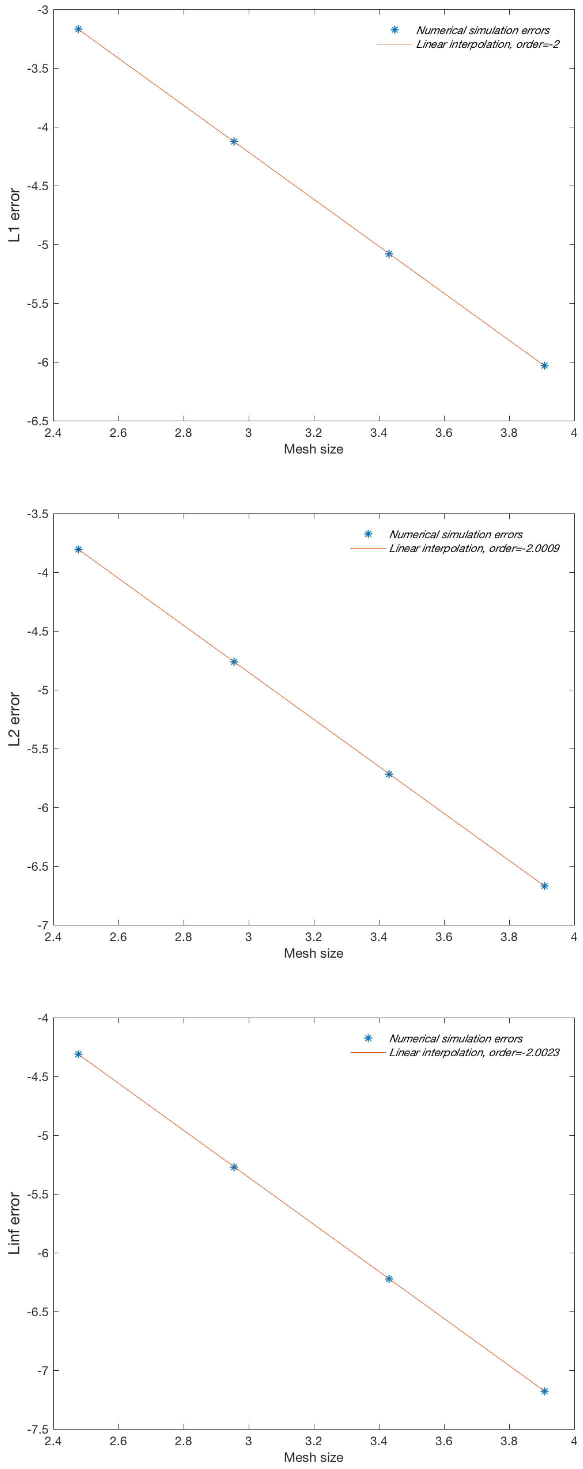

Figure Q. Spatial convergence: L_1, L_2, and L_∞ errors of convergence plotted against the grid size in $\log_{10}$ scale obtained with the FTCS scheme for 1D diffusion for periodic boundary conditions. $D_a = 0.1$. Initial (for the coarsest grid) time step number: 50. Grid sizes: 100, 300, 900, 2700 and 8100 points

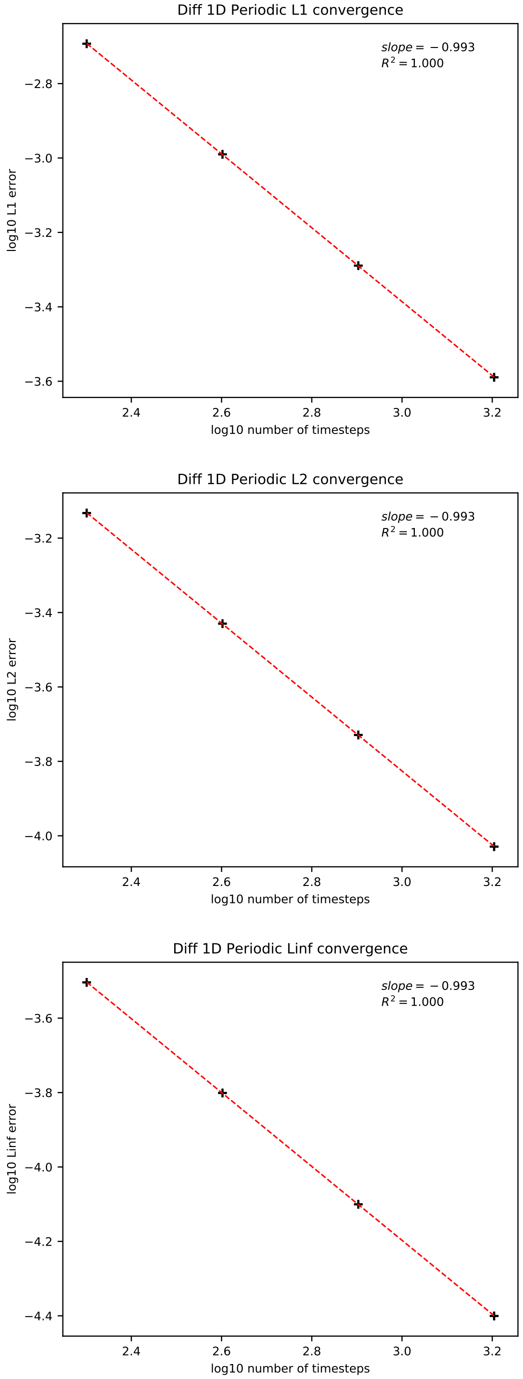

Figure R. Time convergence: L_1, L_2, and L_∞ errors of convergence plotted against the number of time steps in $\log_{10}$ scale obtained with the FTCS scheme for 1D diffusion for periodic boundary conditions. $D_a = 0.1$. Initial time step number: 100. Fixed grid size: 10 points

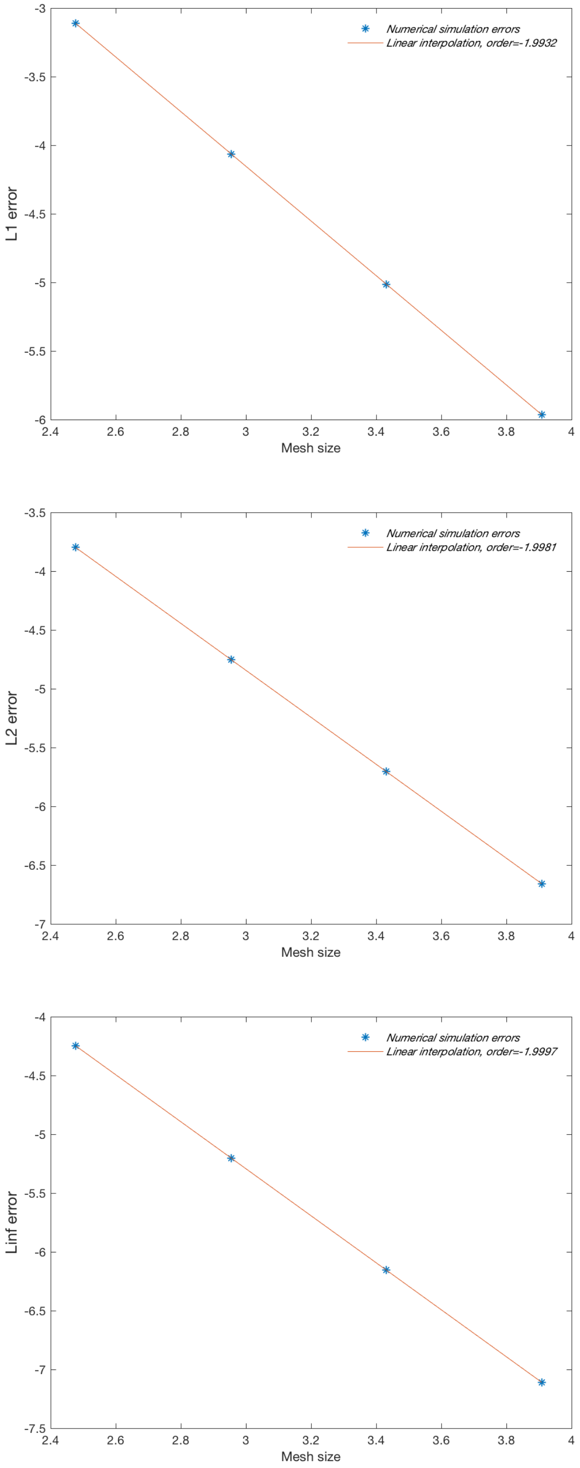

Figure S. Spatial convergence: L_1, L_2, and L_∞ errors of convergence plotted against the grid size in $\log_{10}$ scale obtained with the Crank-Nicolson scheme for 1D diffusion for periodic boundary conditions. Initial $D_a = 0.1$ Fixed time step number: 50. Grid sizes: 100, 300, 900, 2700 and 8100 points

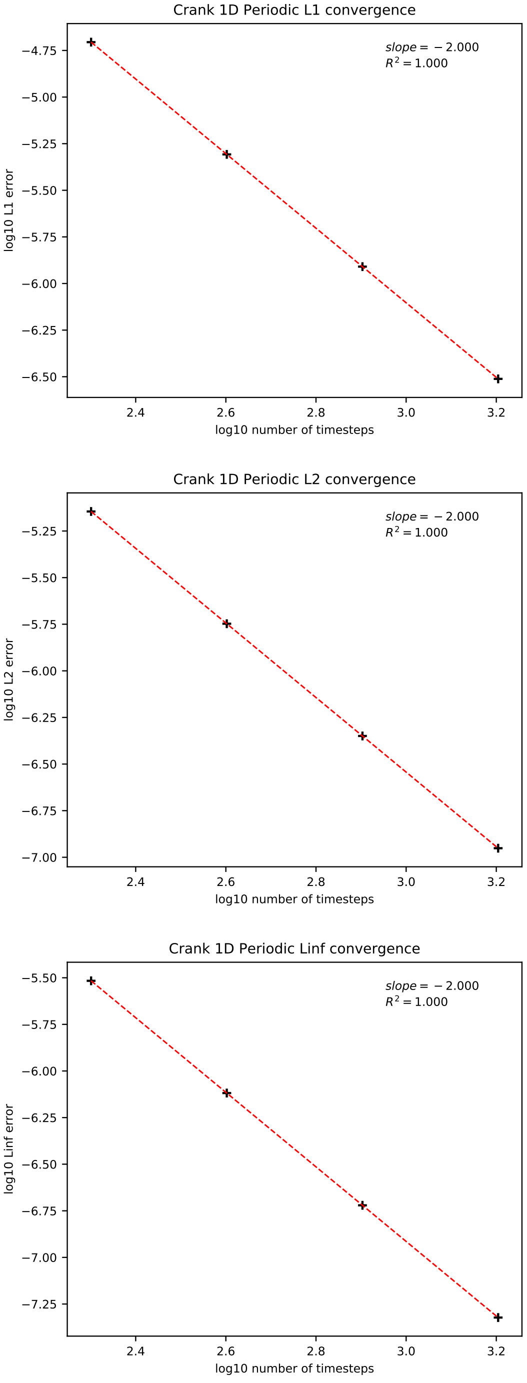

Figure T. Time convergence: L_1, L_2, and L_∞ errors of convergence plotted against the number of time steps in $\log_{10}$ scale obtained with the Crank-Nicolson scheme for 1D diffusion for periodic boundary conditions. $D_a = 0.1$. Initial time step number: 100. Fixed grid size: 10 points

2D

In this set of test cases, we are solving the following equation:

$$\frac{\partial C}{\partial t} - D(\frac{\partial C}{\partial x^2} + \frac{\partial C}{\partial y^2}) = 0 \tag{7}$$

We first tested the forward-time centered space scheme (FTCS), for which the adimensionalized diffusion constant $D_a = \frac{D\Delta t}{\Delta x^2}$ was set to be equal to 0.1, and then the alternating direction implicit (ADI) method scheme, that is unconditionally L_2 stable, for which we assessed the order of convergence in space without adapting the time step.

Dirichlet boundary conditions We considered a Gaussian centered in the middle of the domain as an initial condition:

$$C(0, x, y) = \exp\left(\frac{-(x-0.5)^2 + (y-0.5)^2}{10}\right) \tag{8}$$

and imposed the value of the field to be 0 at the boundaries. The following pictures show the error plots in log scale for both schemes.

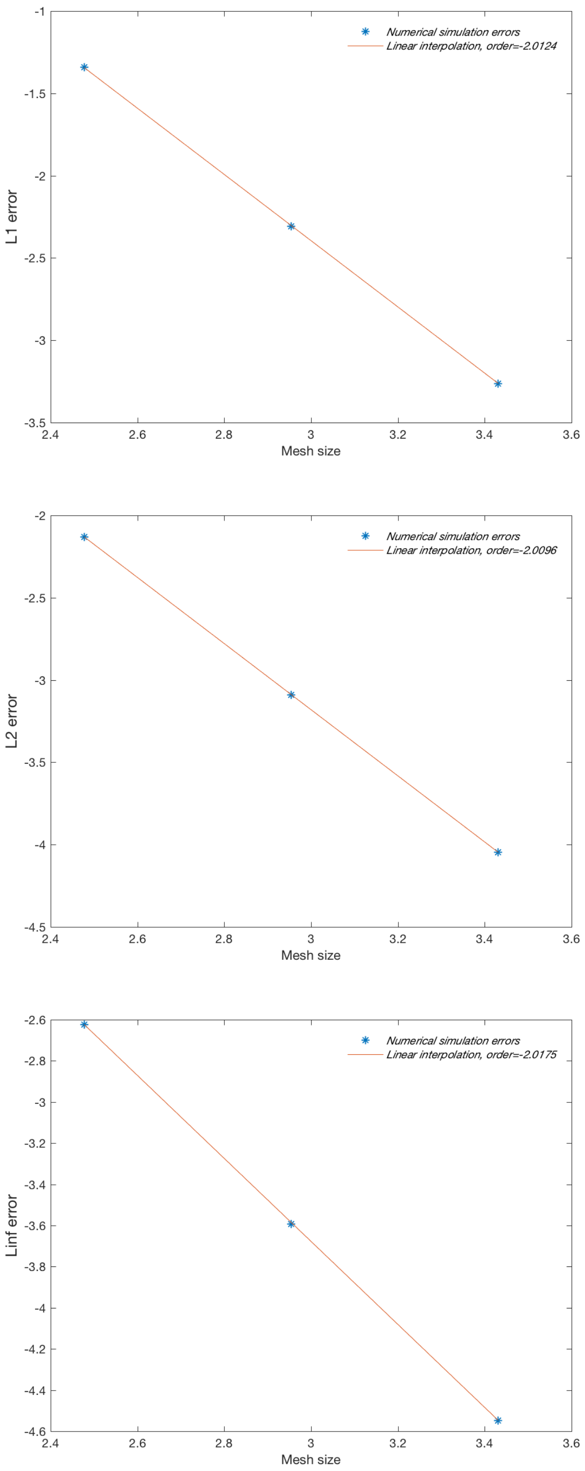

Figure U. Spatial convergence: L_1, L_2, and L_∞ errors of convergence plotted against the grid size in $\log_{10}$ scale obtained with FTCS scheme for 2D diffusion with Dirichlet (0 at the boundaries) boundary conditions. Initial (for the coarsest grid) time step number: 50. $D_a = 0.05$ Grid sizes: 10×10, 30×30, 90×90, 270×270 points

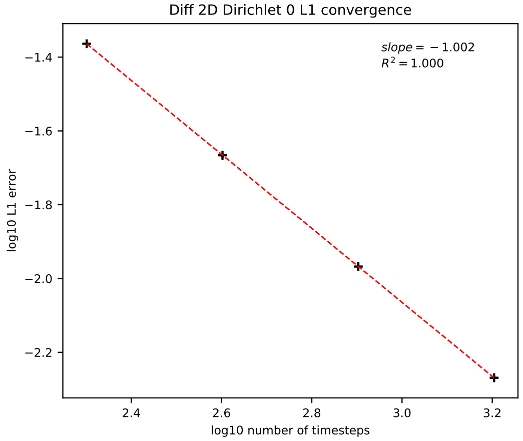

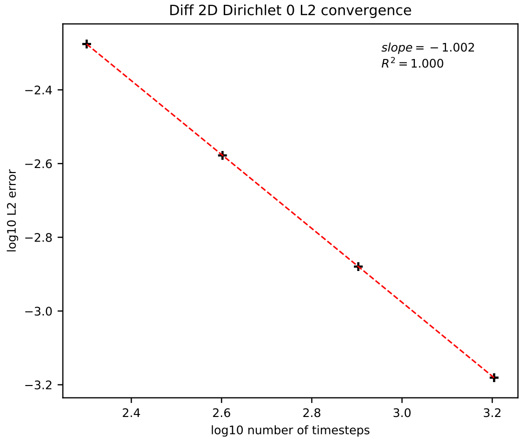

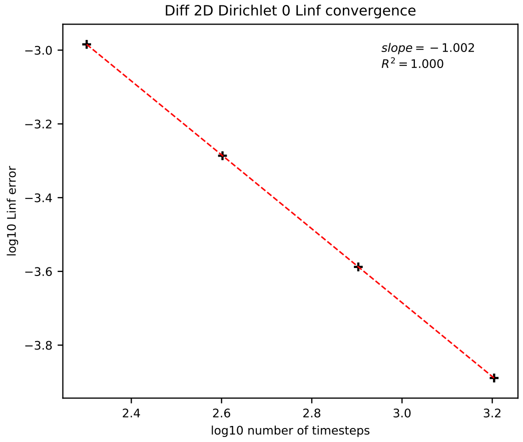

Figure V. Time convergence: L_1, L_2, and L_∞ errors of convergence plotted against the number of time steps in $\log_{10}$ scale obtained with the FTCS scheme for 2D diffusion with Dirichlet (0 at the boundaries) boundary conditions. $D_a = 0.1$. Initial time step number: 100. Fixed grid size: 10 points

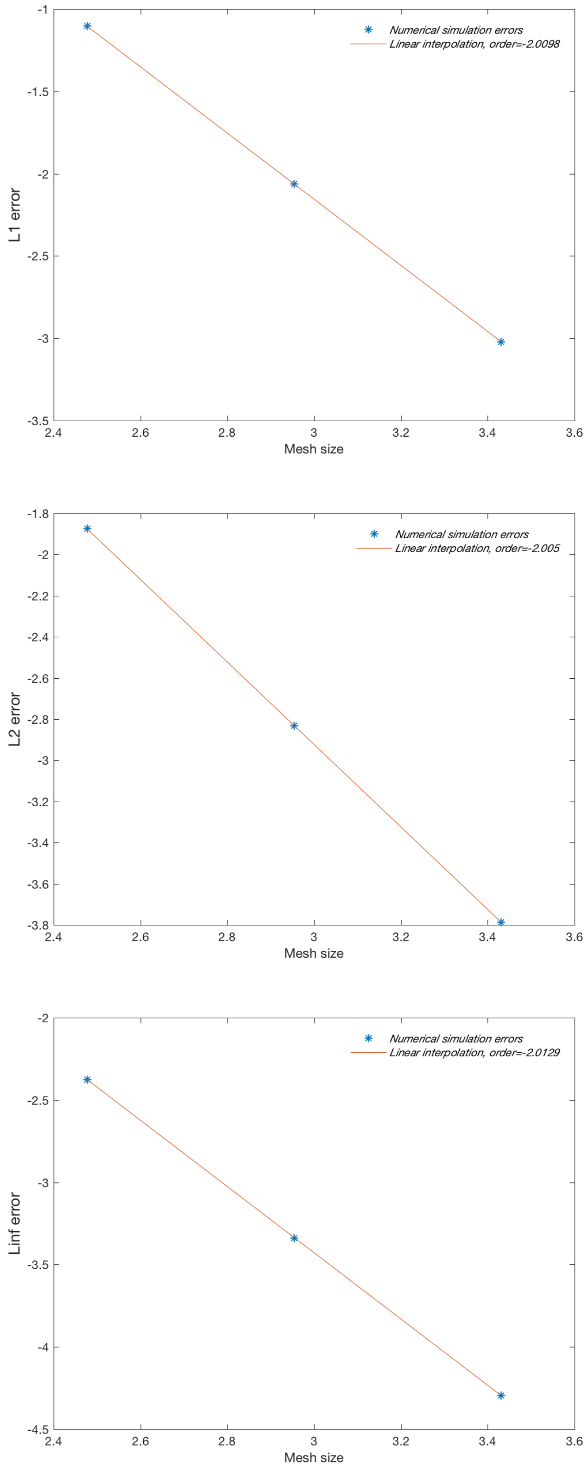

Figure W. Spatial convergence: L_1, L_2, and L_∞ errors of convergence plotted against the grid size in $\log_{10}$ scale obtained with ADI scheme for 2D diffusion with Dirichlet (0 at the boundaries) boundary conditions. Fixed time step number: 50. Grid sizes: 10×10, 30×30, 90×90, 270×270 points

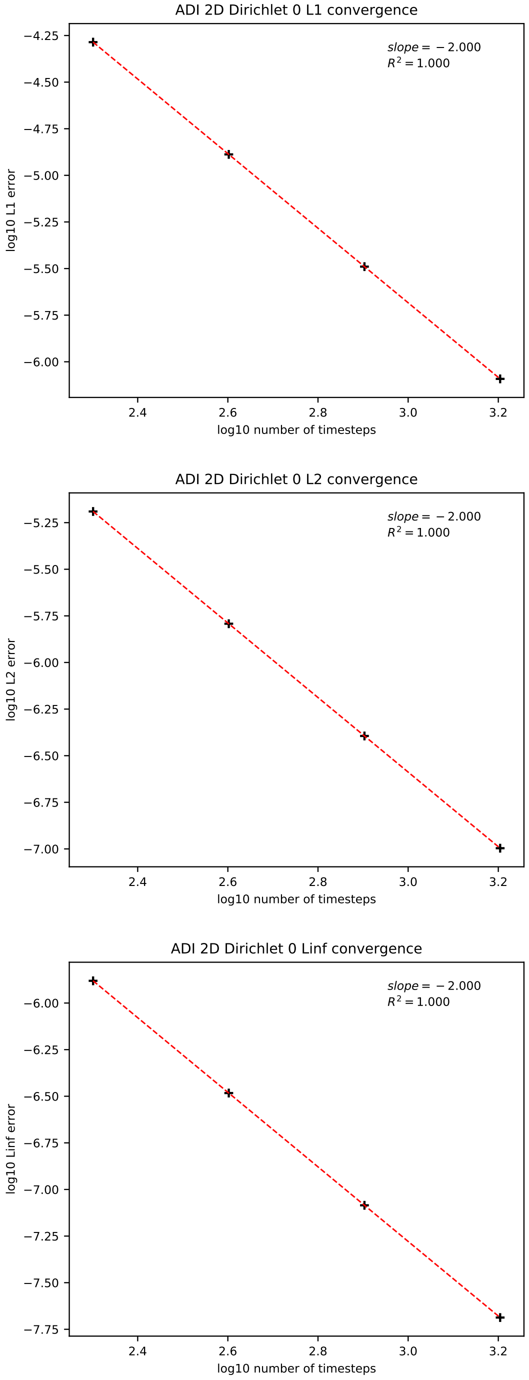

Figure X. Time convergence: L_1, L_2, and L_∞ errors of convergence plotted against the number of time steps in $\log_{10}$ scale obtained with the ADI scheme for 2D diffusion with Dirichlet (0 at the boundaries) boundary conditions. Initial $D_a = 0.1$. Initial time step number: 100. Fixed grid size: 10 points

Neumann boundary conditions. We considered a Gaussian centered in the

middle of the domain as an initial condition and imposed no flux at the boundaries.

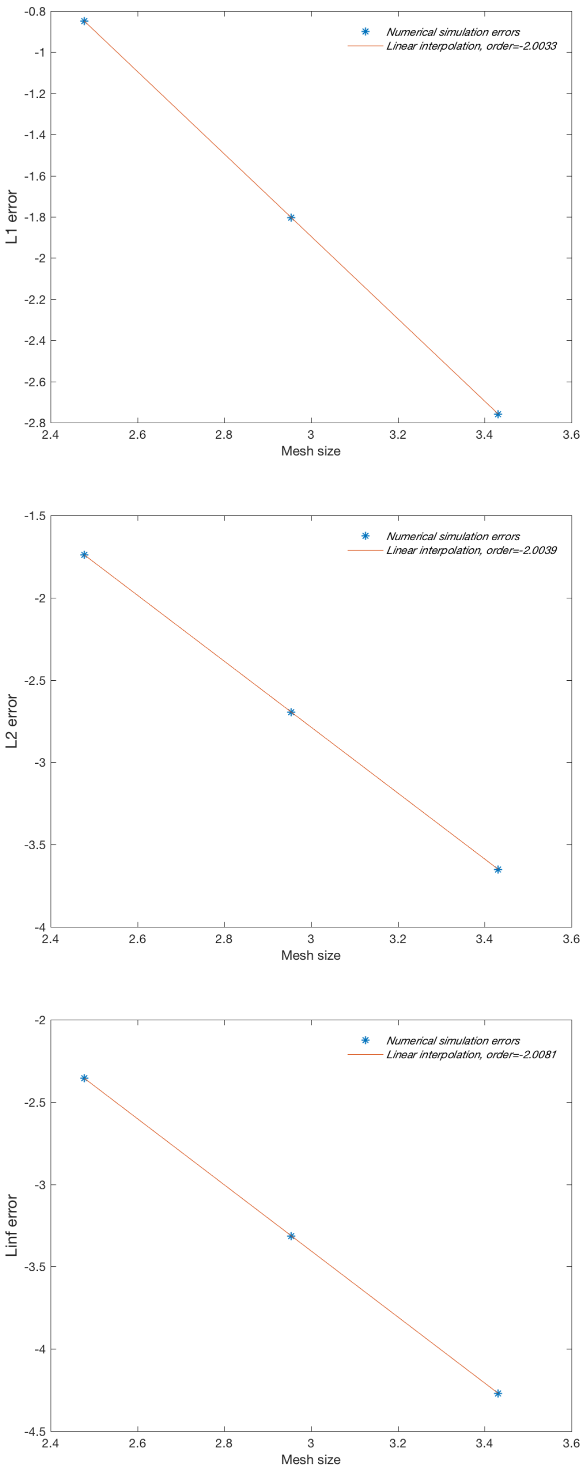

Figure Y. Spatial convergence: L_1, L_2, and L_∞ errors of convergence plotted against the grid size in $\log_{10}$ scale obtained with FTCS scheme for 2D diffusion with no flux boundary conditions. Initial (for the coarsest grid) time step number: 50. $D_a = 0.05$ Grid sizes: 10×10, 30×30, 90×90, 270×270 points

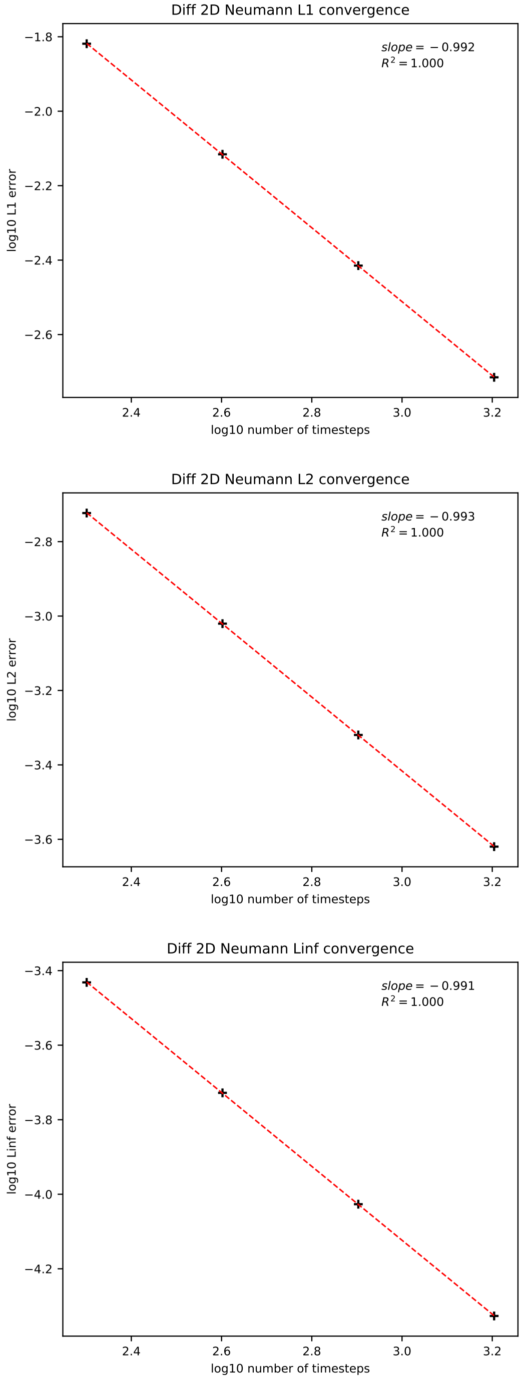

Figure Z. Time convergence: L_1, L_2, and L_∞ errors of convergence plotted against the number of time steps in $\log_{10}$ scale obtained with the FTCS scheme for 2D diffusion with Von-Neumann boundary conditions. $D_a = 0.1$. Initial time step number: 100. Fixed grid size: 10 points

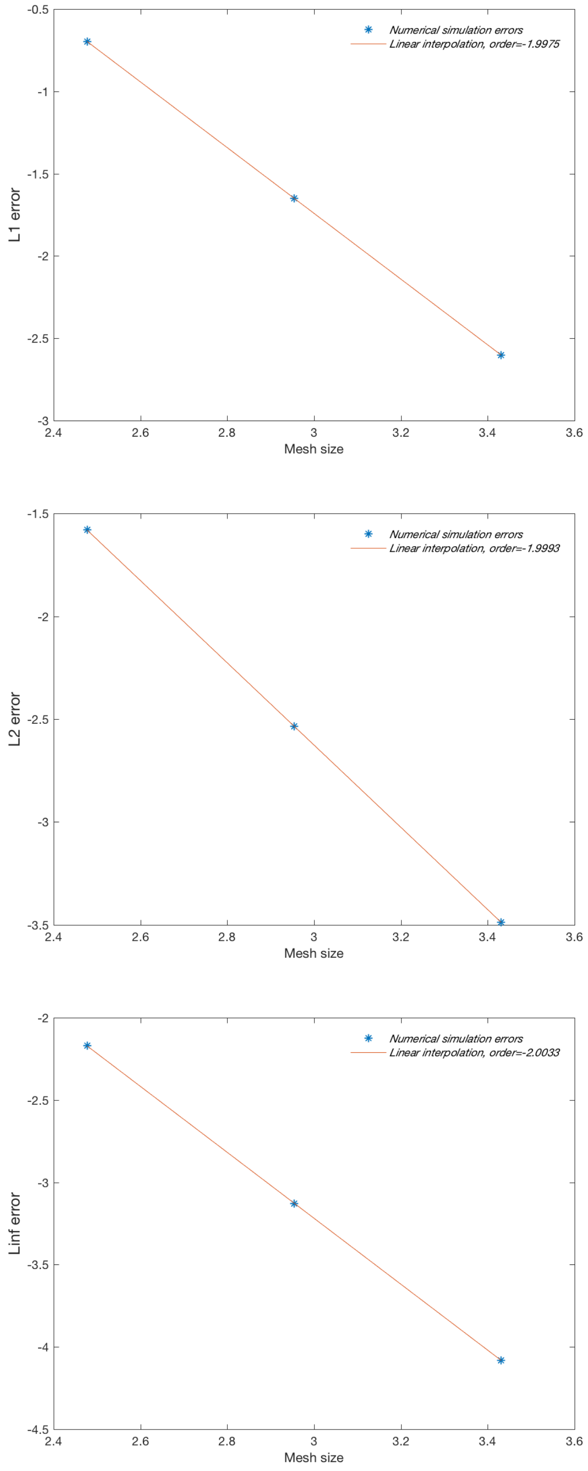

Figure AA. Spatial convergence: L_1, L_2, and L_∞ errors of convergence plotted against the grid size in $\log_{10}$ scale obtained with ADI scheme for 2D diffusion with no flux boundary conditions. Fixed time step number: 50. Initial $D_a = 0.05$ Grid sizes: 10×10, 30×30, 90×90, 270×270 points

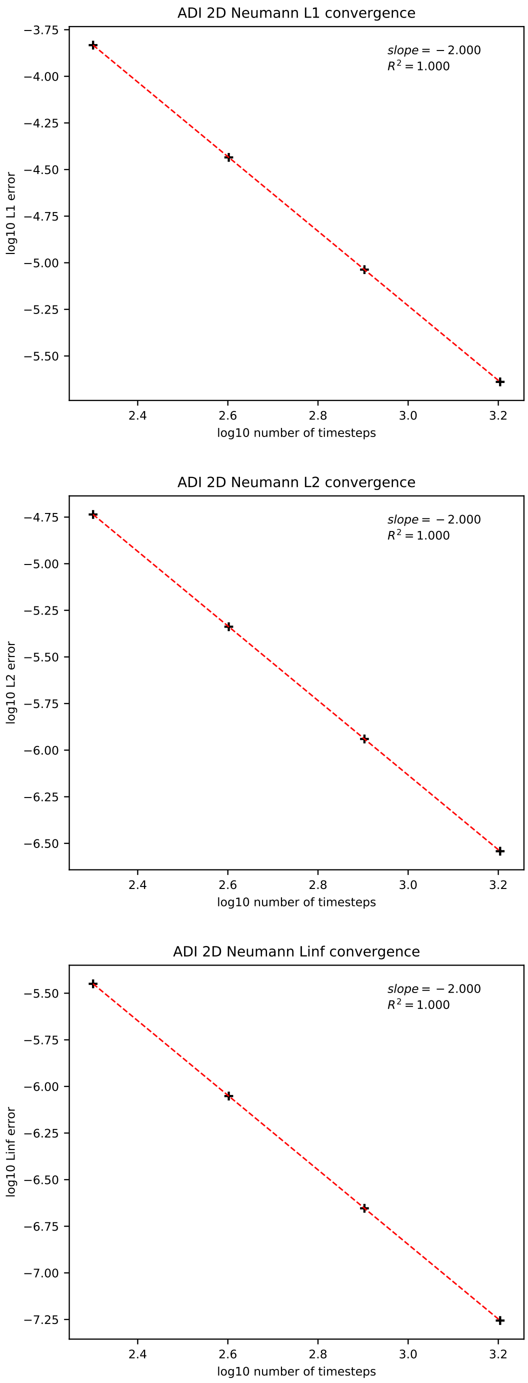

Figure AB. Time convergence: L_1, L_2, and L_∞ errors of convergence plotted against the number of time steps in $\log_{10}$ scale obtained with the ADI scheme for 2D diffusion with Von-Neumann boundary conditions. $D_a = 0.1$. Initial time step number: 100. Fixed grid size: 10 points

Periodic boundary conditions. In this test case, we consider the regular

symmetrical initial condition defined as follows:

$$C(0,x) = \exp\left(-\frac{(x-0.5)^2}{1000}\right) \tag{9}$$

We then solved the diffusion equation with periodic boundary conditions.

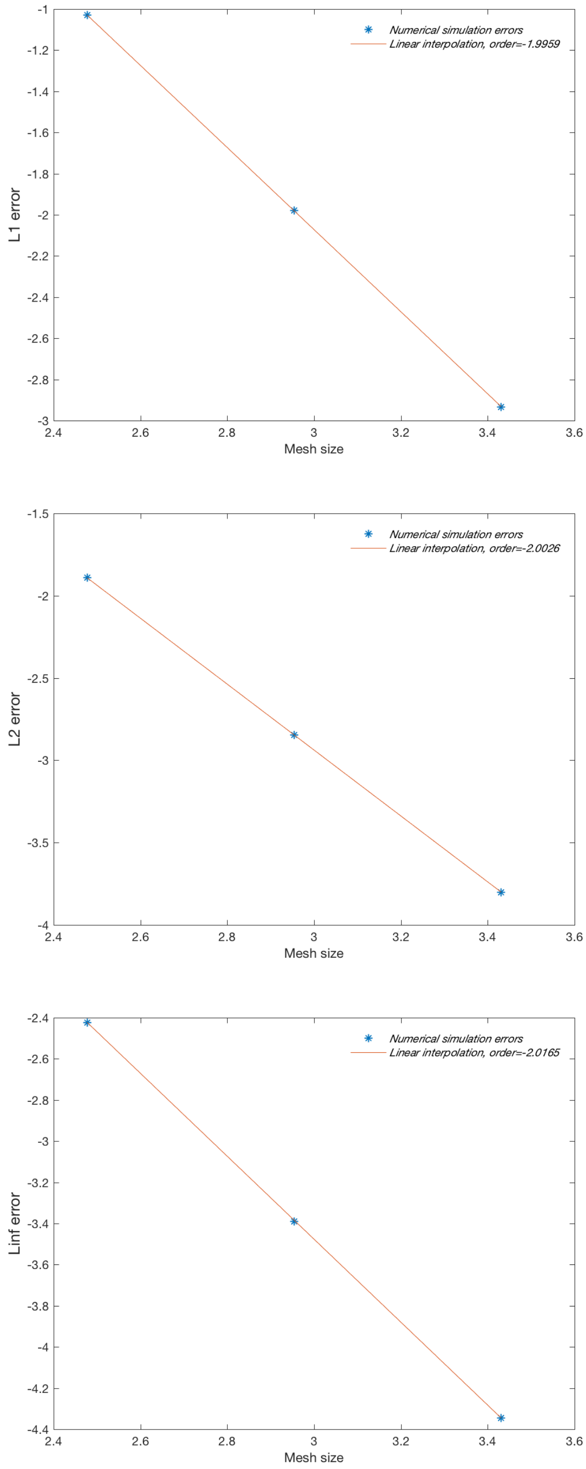

Figure AC. Spatial convergence: L_1, L_2, and L_∞ errors of convergence plotted against the grid size in $\log_{10}$ scale obtained with FTCS scheme for 2D diffusion with periodic boundary conditions. Initial (for the coarsest grid) time step number: 20. $D_a = 0.05$ Grid sizes: 10×10, 30×30, 90×90, 270×270 points

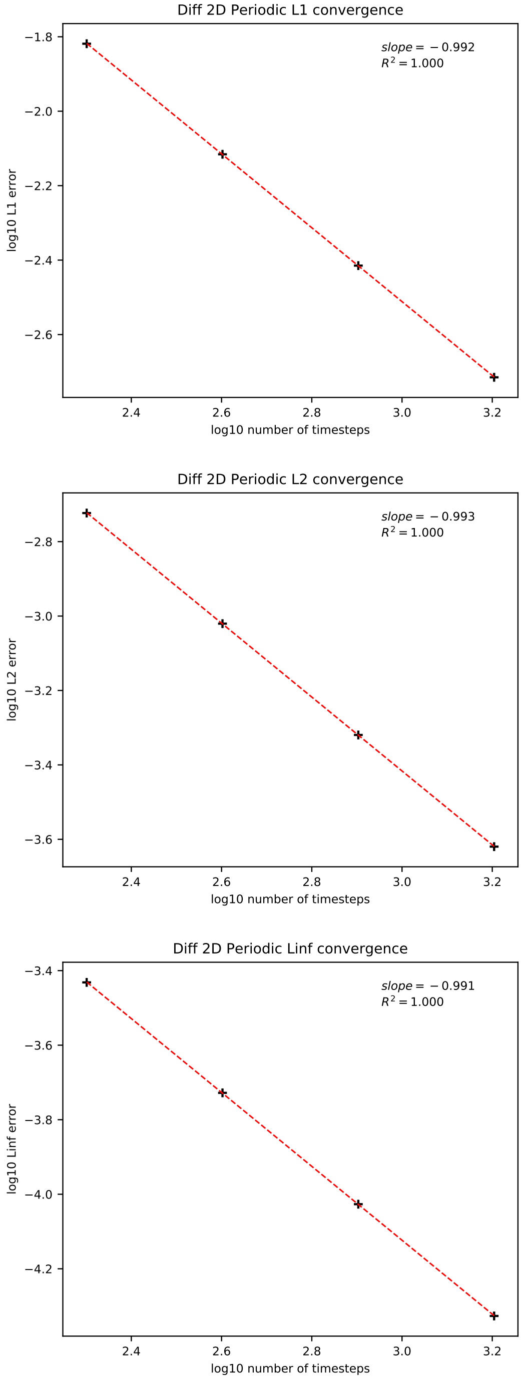

Figure AD. Time convergence: L_1, L_2, and L_∞ errors of convergence plotted against the number of time steps in $\log_{10}$ scale obtained with the FTCS scheme for 2D diffusion with periodic boundary conditions. $D_a = 0.1$. Initial time step number: 100. Fixed grid size: 10 points

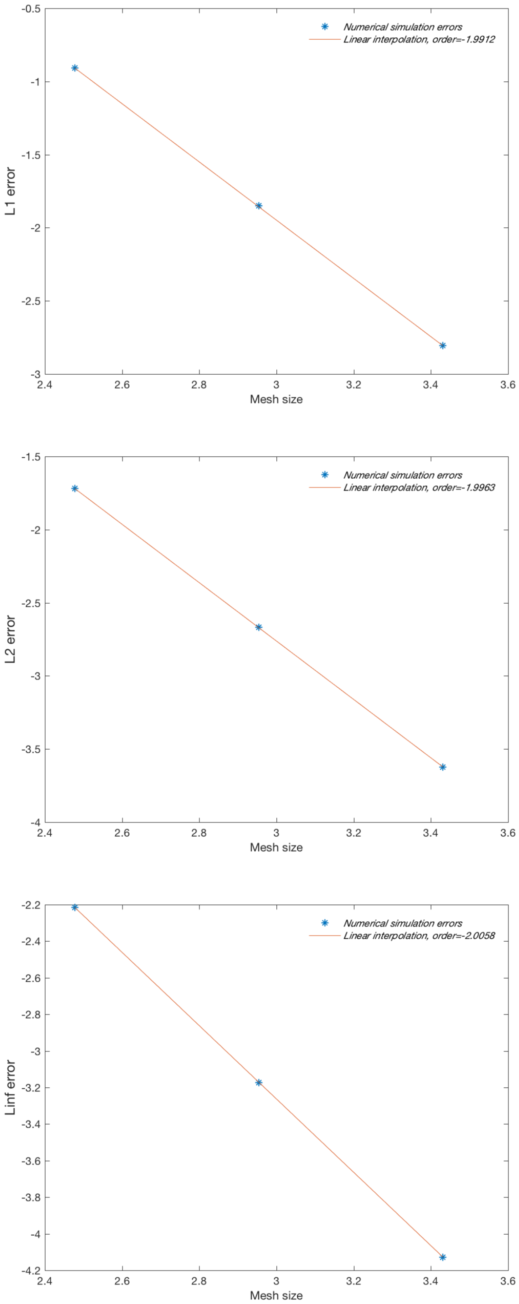

Figure AE. Spatial convergence: L_1, L_2, and L_∞ errors of convergence plotted against the grid size in $\log_{10}$ scale obtained with ADI scheme for 2D diffusion with periodic boundary conditions. Fixed time step number: 20. Initial $D_a = 0.05$ Grid sizes: 10×10, 30×30, 90×90, 270×270 points

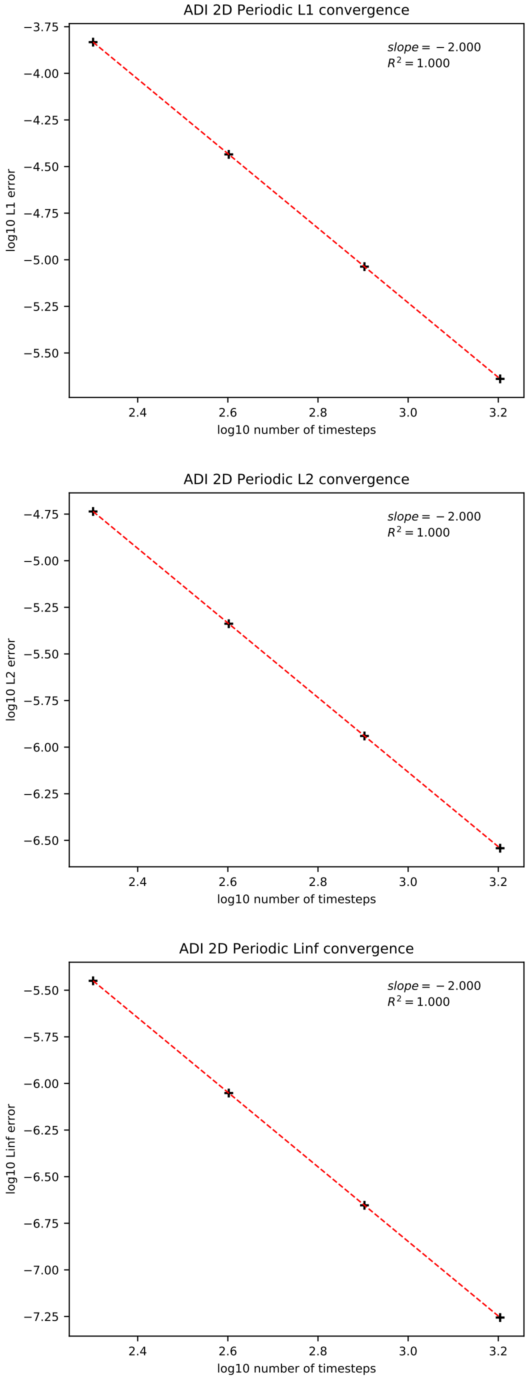

Figure AF. Time convergence: L_1, L_2, and L_∞ errors of convergence plotted against the number of time steps in $\log_{10}$ scale obtained with the ADI scheme for 2D diffusion with periodic boundary conditions. $D_a = 0.1$. Initial time step number: 100. Fixed grid size: 10 points

The following figures show the initial and final distributions for both schemes:

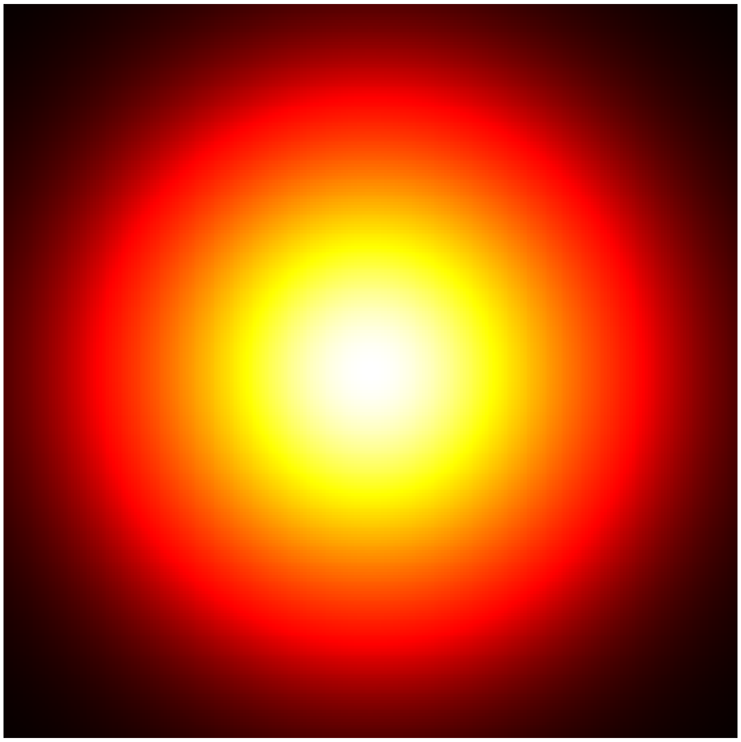

Figure AG. Initial distribution before running diffusion solver

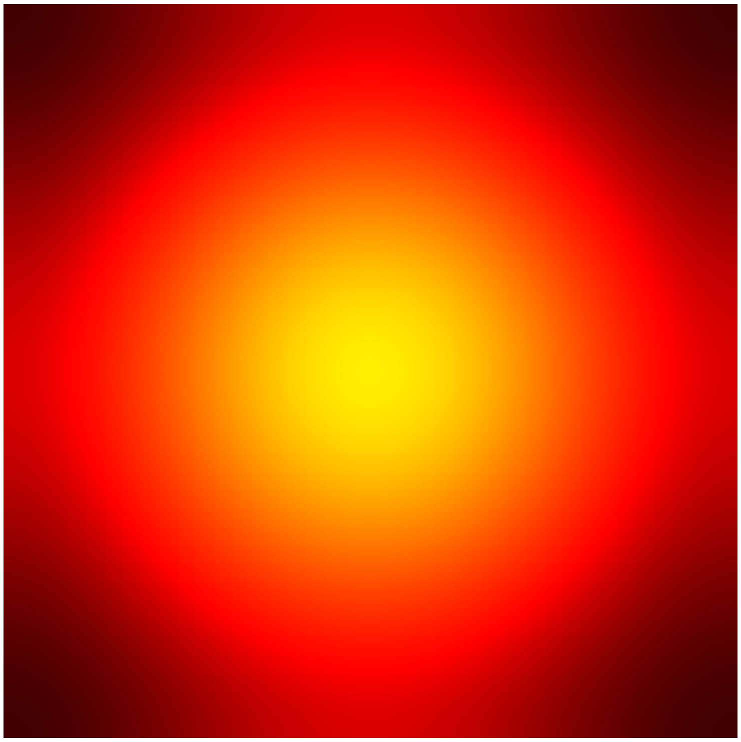

Figure AH. Final distribution after simulation using FTCS scheme for 2D diffusion under periodic boundary conditions

3D

In this set of test cases, we are solving the following equation:

$$\frac{\partial C}{\partial t} - D(\frac{\partial C}{\partial x^2} + \frac{\partial C}{\partial y^2} + \frac{\partial C}{\partial z^2}) = 0 \tag{10}$$

We tested the forward-time centered space scheme (FTCS), for which the adimensionalized diffusion constant $D_a = \frac{D\Delta t}{\Delta x^2}$ was set to be equal to 0.05.

Dirichlet boundary conditions We considered a Gaussian centered in the middle of the domain as an initial condition:

$$C(0, x, y, z) = \exp\left(\frac{-(x-0.5)^2 + (y-0.5)^2 + (z-0.5)^2}{10}\right) \tag{11}$$

and imposed the value of the field to be 0 at the boundaries.

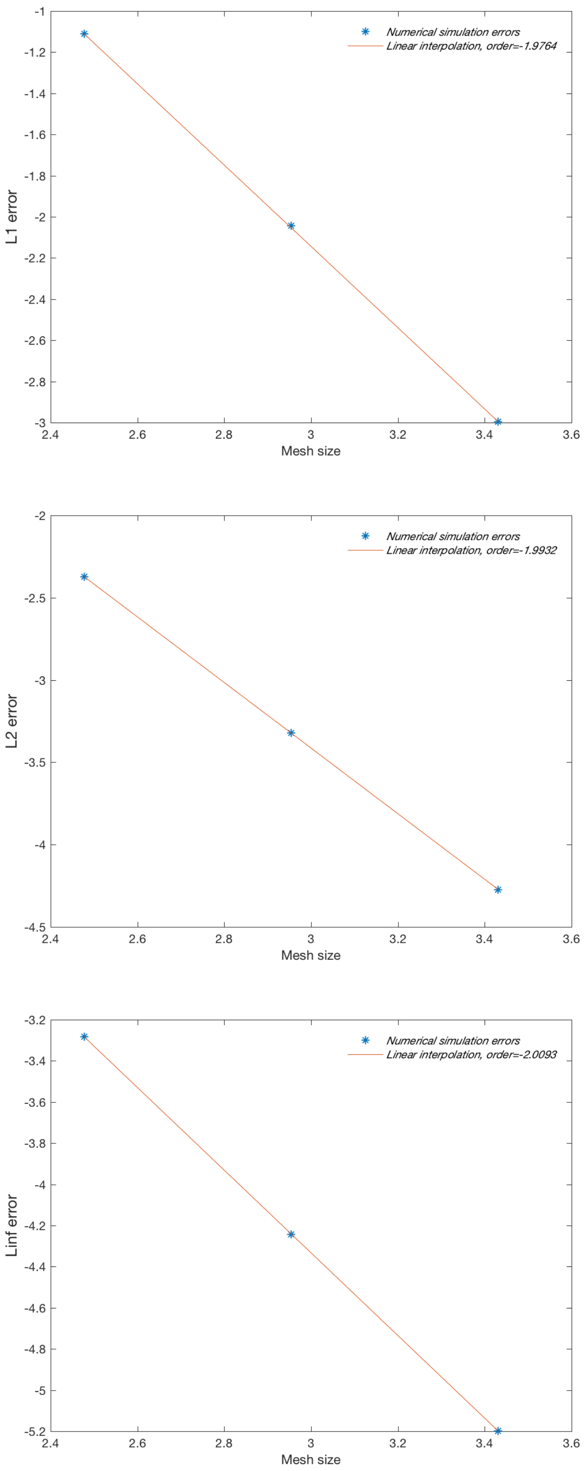

Figure AI. Spatial convergence: L_1, L_2, and L_∞ errors of convergence plotted against the grid size in $\log_{10}$ scale obtained with FTCS scheme for 3D diffusion with Dirichlet (0 on the boundaries) boundary conditions. Initial (for the coarsest grid) time step number: 100. $D_a = 0.05$ Grid sizes: $10 \times 10 \times 10$, $30 \times 30 \times 30$, $90 \times 90 \times 90$, $270 \times 270 \times 270$ points

Neumann boundary conditions. We considered a Gaussian centered in the middle of the domain as an initial condition and imposed no flux at the boundaries.

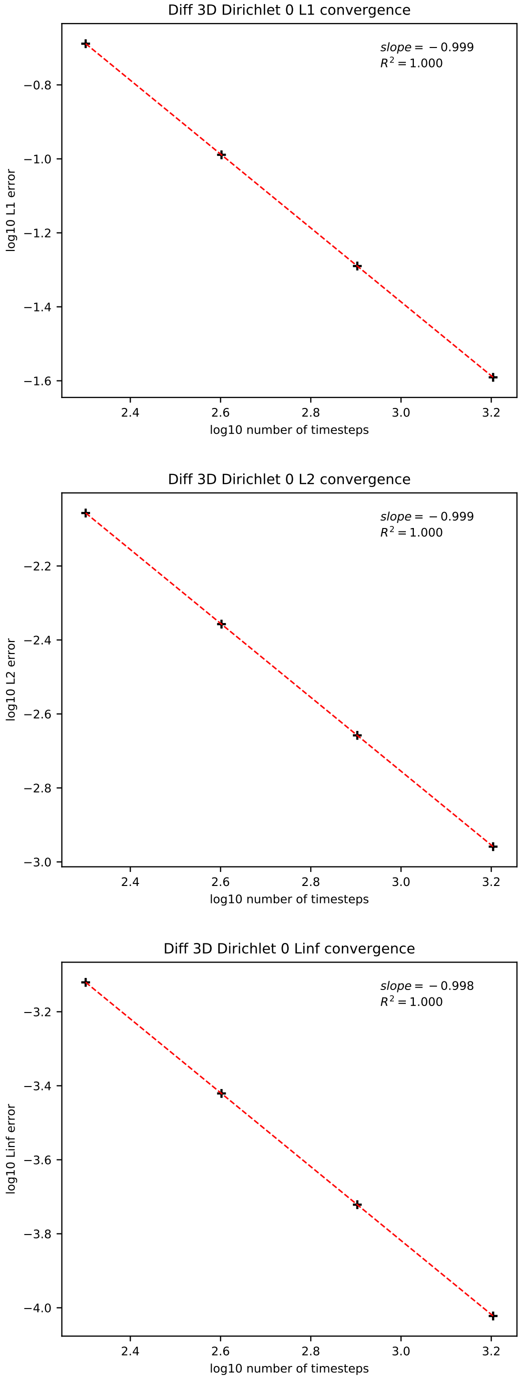

Figure AJ. Time convergence: L_1, L_2, and L_∞ errors of convergence plotted against the number of time steps in $\log_{10}$ scale obtained with the FTCS scheme for 3D diffusion with periodic boundary conditions. $D_a = 0.1$. Initial time step number: 100. Fixed grid size: 10 points

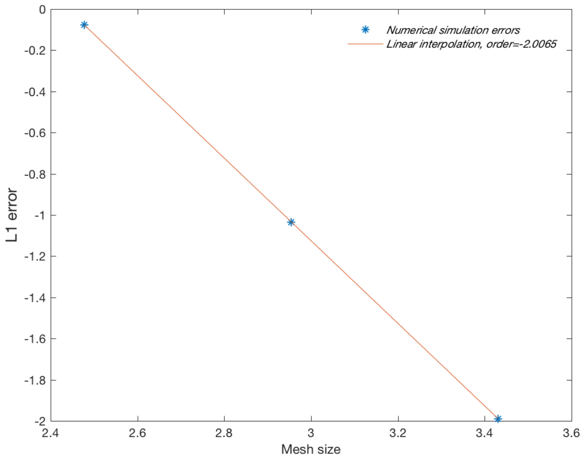

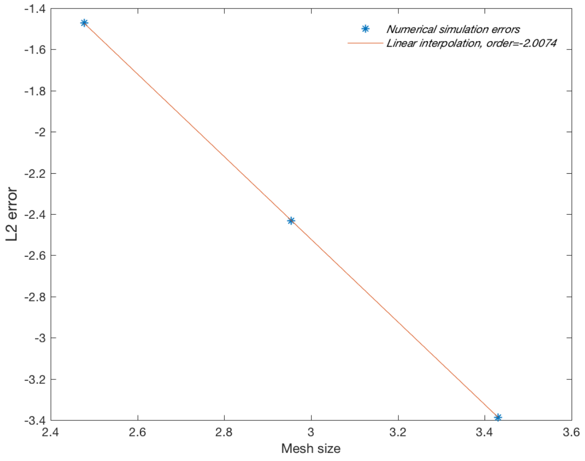

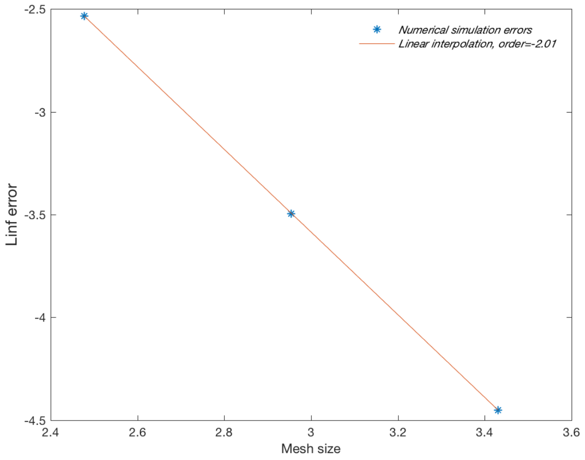

Figure AK. Spatial convergence: L_1, L_2, and L_∞ errors of convergence plotted against the grid size in $\log_{10}$ scale obtained with FTCS scheme for 3D diffusion with no flux boundary conditions. Initial (for the coarsest grid) time step number: 100. $D_a = 0.05$ Grid sizes: $10 \times 10 \times 10$, $30 \times 30 \times 30$, $90 \times 90 \times 90$, $270 \times 270 \times 270$ points

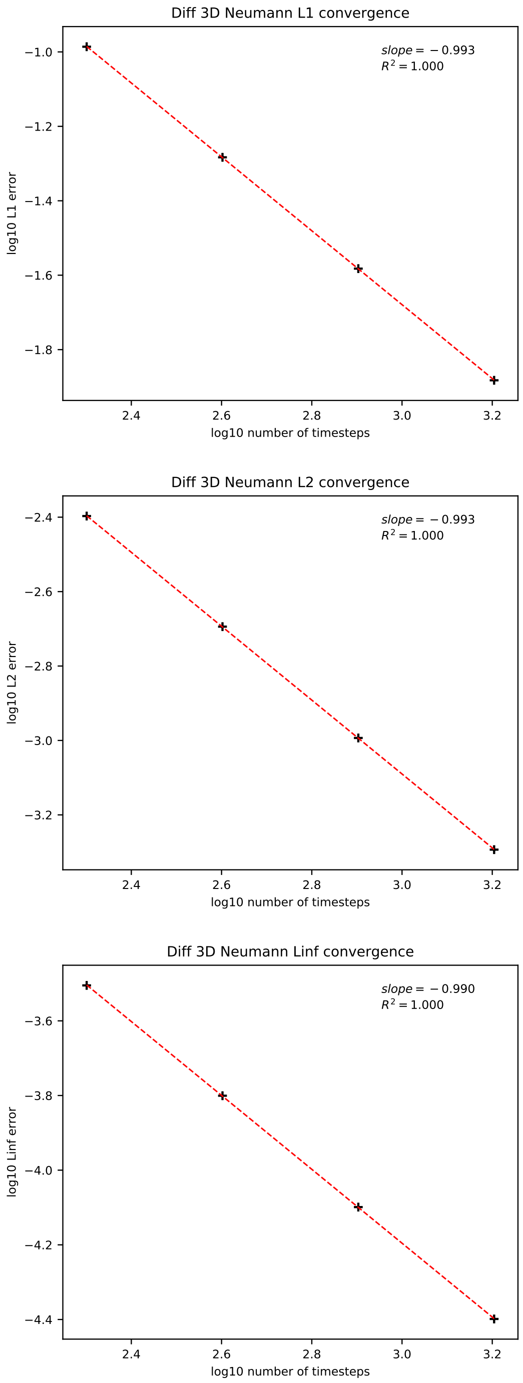

Figure AL. Time convergence: L_1, L_2, and L_∞ errors of convergence plotted against the number of time steps in $\log_{10}$ scale obtained with the FTCS scheme for 3D diffusion with Von-Neumann boundary conditions. $D_a = 0.1$. Initial time step number: 100. Fixed grid size: 10 points

Advection

As theoretically expected, for all dimensions, the estimated order of convergence in space and time was one for the Upwind scheme.

1D

In this set of test cases, we are numerically solving the linear advection equation:

$$\frac{\partial C}{\partial t} + v\frac{\partial C}{\partial x} = 0 \tag{12}$$

We tested the first order Upwind scheme for convergence in space and time. For the convergence in space, the normalized adimensionalized velocity $v_a = \frac{v\Delta t}{\Delta x}$ was set to be equal to 0.1 for the Dirichlet test case and 0.5 for the periodic boundary condition test case. For the convergence in time, it was set to 0.1 for the initial time resolution.

Dirichlet boundary condition on the left, no boundary condition on the right. In this test case, the velocity field is homogeneous and unidirectional, from left to right. The initial density profile follows a regular function whose value is one at the left boundary and 0 at the right boundary. The reason for this is that imposing a discontinuity through the boundary conditions would lead to conservation of this discontinuous front through advection and no hope to get at least order 1 convergence (since the numerical scheme assumes a differentiable field everywhere on the domain). For the density profile, we chose a Gaussian function defined as follows:

$$C(0, x) = \exp\left(-\frac{x^2}{2}\right) \tag{13}$$

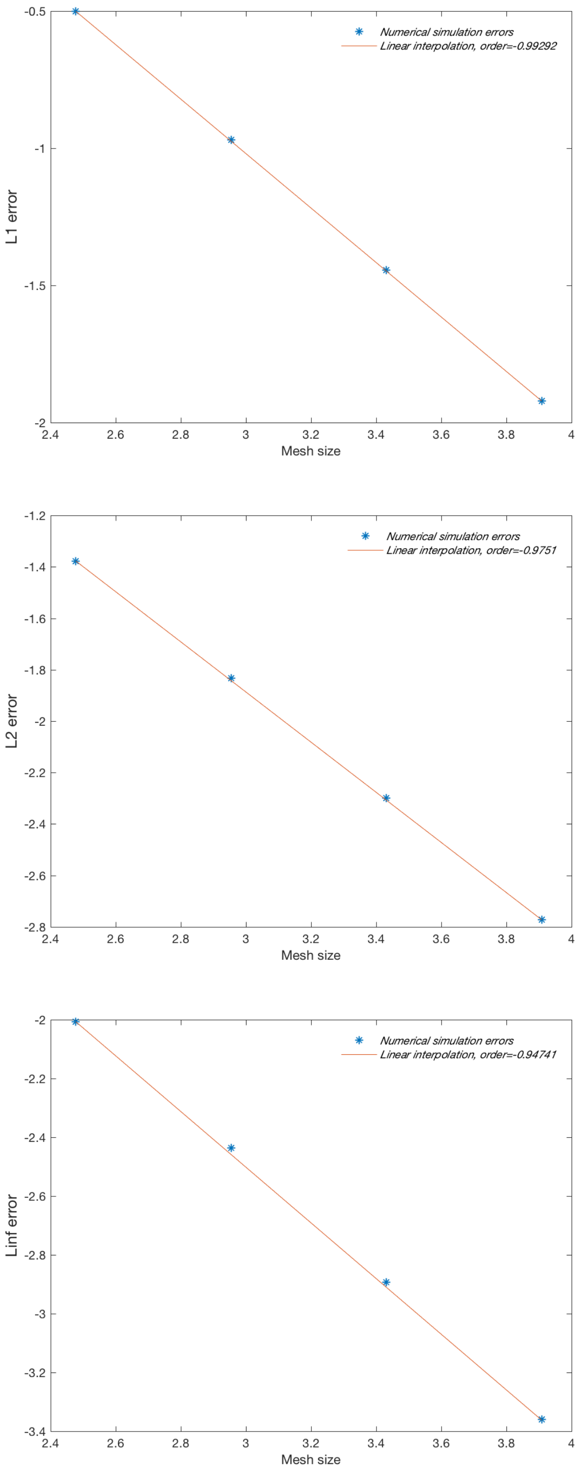

Figure AM. Spatial convergence: L_1, L_2, and L_∞ errors of convergence plotted against the grid size in $\log_{10}$ scale obtained with the Upwind scheme for 1D advection with Dirichlet boundary condition on the left, no boundary condition on the right. $v_a = 0.1$. Initial (for the coarsest grid) time step number: 600. Grid sizes: 100, 300, 900, 2700 and 8100 points

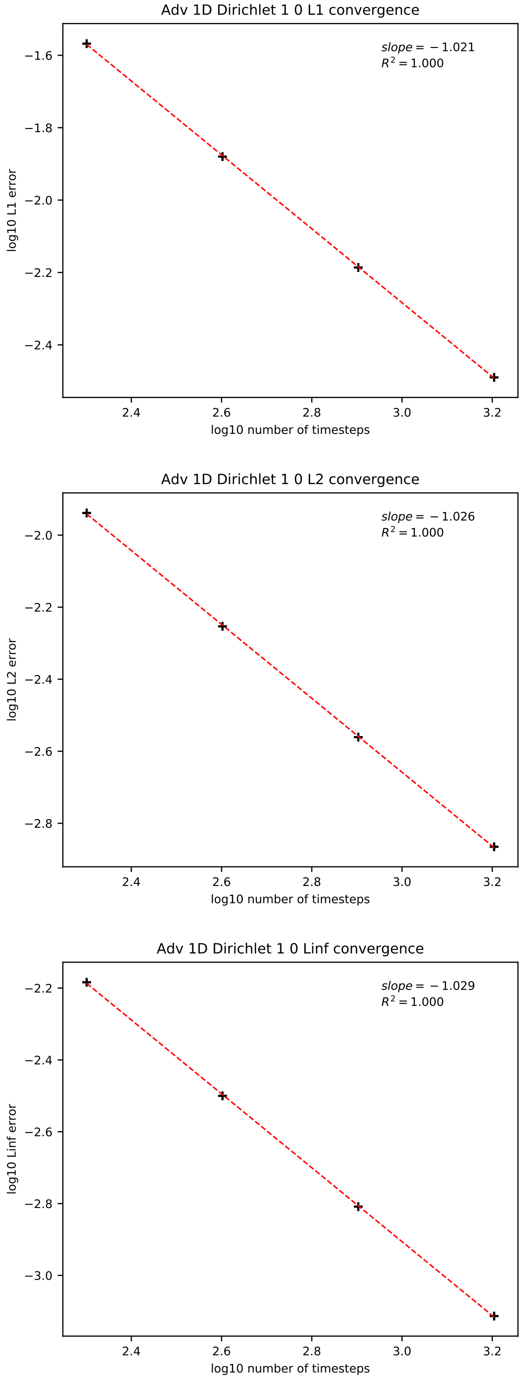

Figure AN. Time convergence: L_1, L_2, and L_∞ errors of convergence plotted against the number of time steps in $\log_{10}$ scale obtained with the Upwind scheme for 3D advection with Dirichlet boundary conditions on the left, no boundary condition on the right. $v_a = 0.1$. Initial time step number: 100. Fixed grid size: 10 points

Periodic boundary conditions In this test case, we consider the regular

symmetrical initial condition defined as follows:

$$C(0, x) = \exp\left(-\frac{(x - 0.5)^2}{1000}\right) \tag{14}$$

The velocity field is still uniform and unidirectional from left to right.

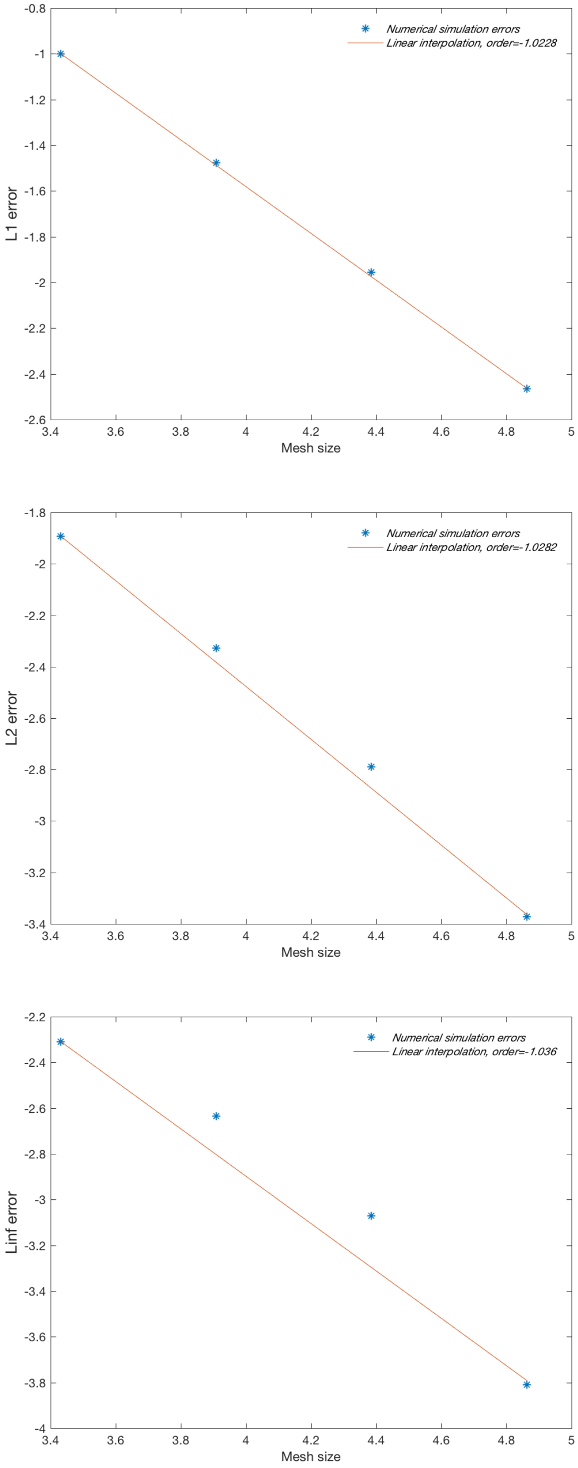

Figure AO. Spatial convergence: L_1, L_2, and L_∞ errors of convergence plotted against the grid size in $\log_{10}$ scale obtained with the Upwind scheme for 1D advection with periodic boundary condition on the left, no boundary condition on the right. $v_a = 0.5$. Initial (for the coarsest grid) time step number: 900. Grid sizes: 900, 2700, 00, 8100 and 24300 points

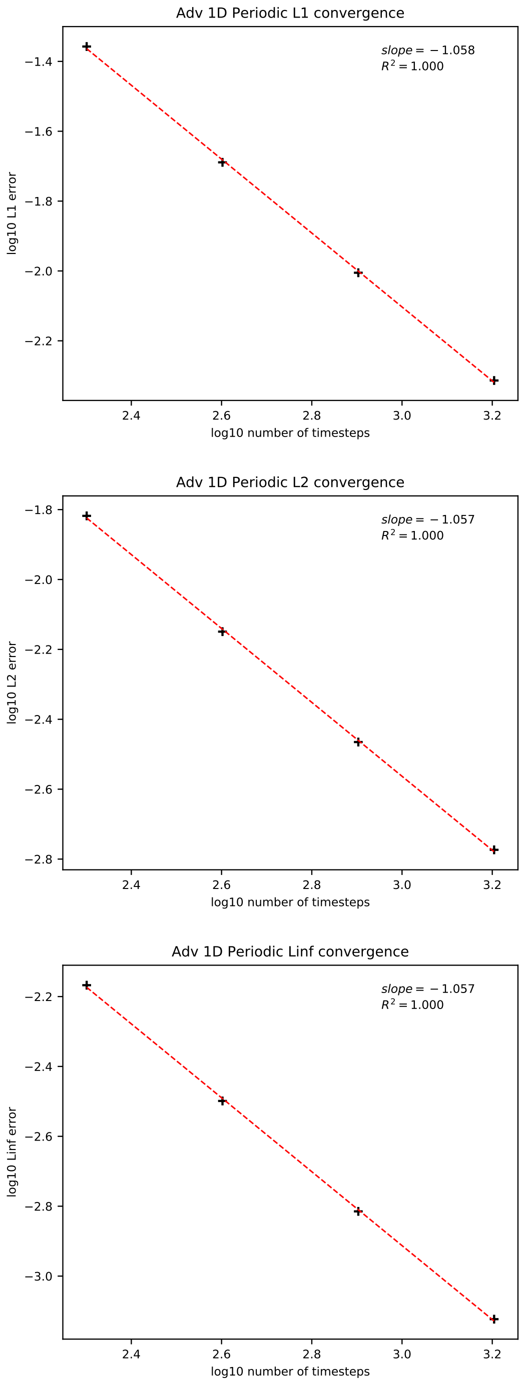

Figure AP. Time convergence: L_1, L_2, and L_∞ errors of convergence plotted against the number of time steps in $\log_{10}$ scale obtained with the Upwind scheme for 1D advection with periodic boundary conditions on the left, no boundary condition on the right. $v_a = 0.1$. Initial time step number: 100. Fixed grid size: 10 points

2D

In this set of test cases, we are numerically solving the linear advection equation:

$$\frac{\partial C}{\partial t} + v_x \frac{\partial C}{\partial x} + v_y \frac{\partial C}{\partial y} = 0 \tag{15}$$

We tested the first order Upwind scheme for convergence in space and time. For the convergence in space, the normalized adimensionalized velocity $v_a = \frac{v \Delta t}{\Delta x}$ was set to be equal to 0.45 for the periodic boundary condition test case. For the convergence in time, it was set to 0.1 for the initial time resolution.

Periodic boundary conditions Here, we consider the regular symmetrical initial condition defined as follows:

$$C(0, x, y) = \exp\left(-\frac{(x-0.5)^2 + (y-0.5)^2}{10}\right) \tag{16}$$

The velocity field is still uniform and unidirectional from left to right.

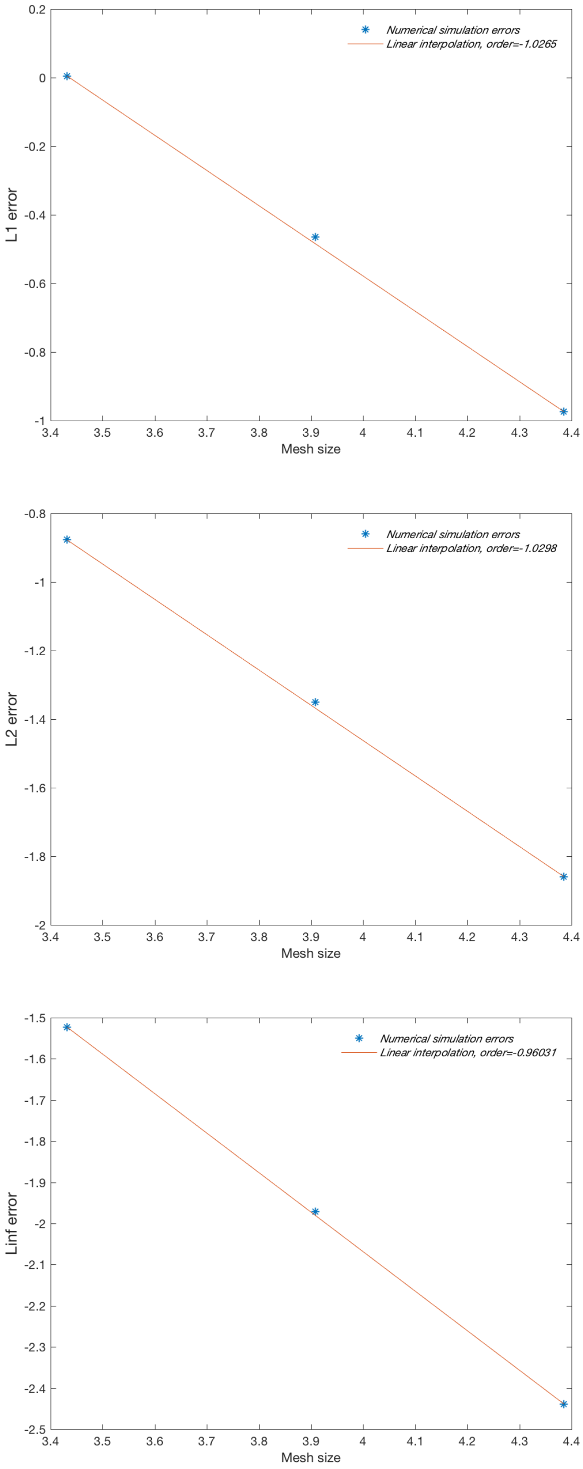

Figure AQ. Spatial convergence: L_1, L_2, and L_∞ errors of convergence plotted against the grid size in $\log_{10}$ scale obtained with the Upwind scheme for 2D advection with periodic boundary condition on the left, no boundary condition on the right. $v_a = 0.45$. Initial (for the coarsest grid) time step number: 20. Grid sizes: 90×90, 270×270, 810×810 and 2430×2430 points

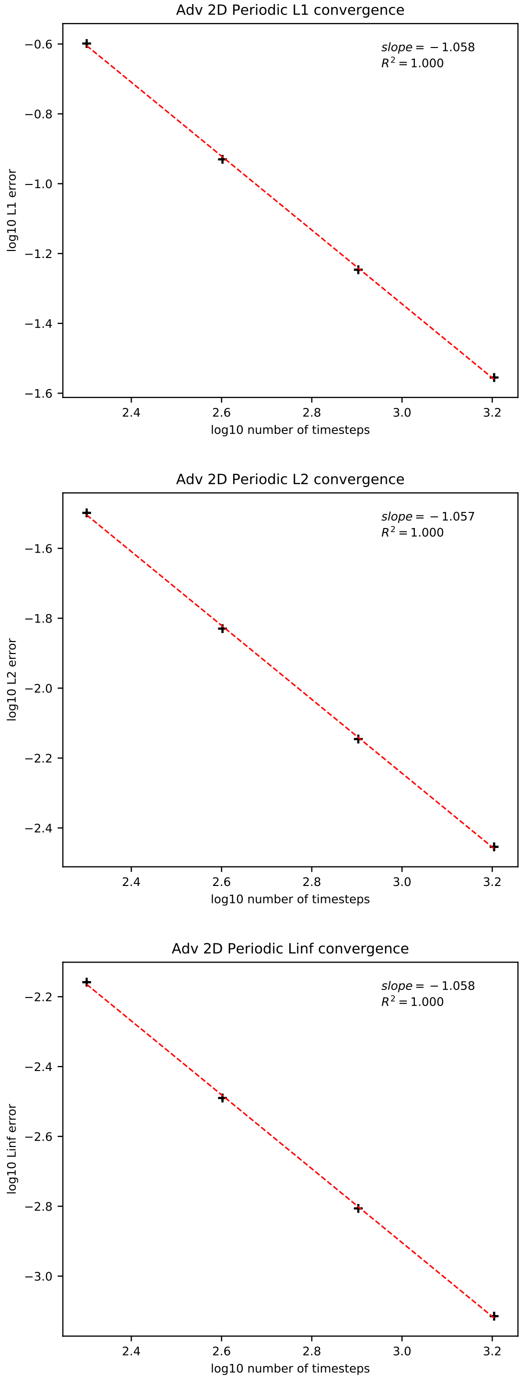

Figure AR. Time convergence: L_1, L_2, and L_∞ errors of convergence plotted against the number of time steps in $\log_{10}$ scale obtained with the Upwind scheme for 2D advection with periodic boundary conditions on the left, no boundary condition on the right. $v_a = 0.1$. Initial time step number: 100. Fixed grid size: 10 points

The following pictures show the initial and final distributions, showing how the

initial distribution is transported on almost an entire period (from left to right).

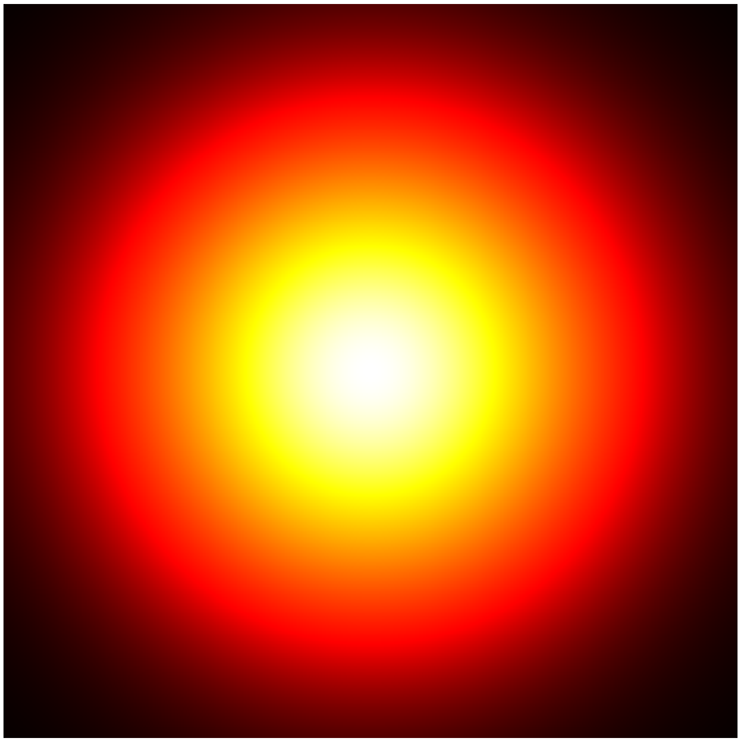

Figure AS. Initial distribution before running advection solver

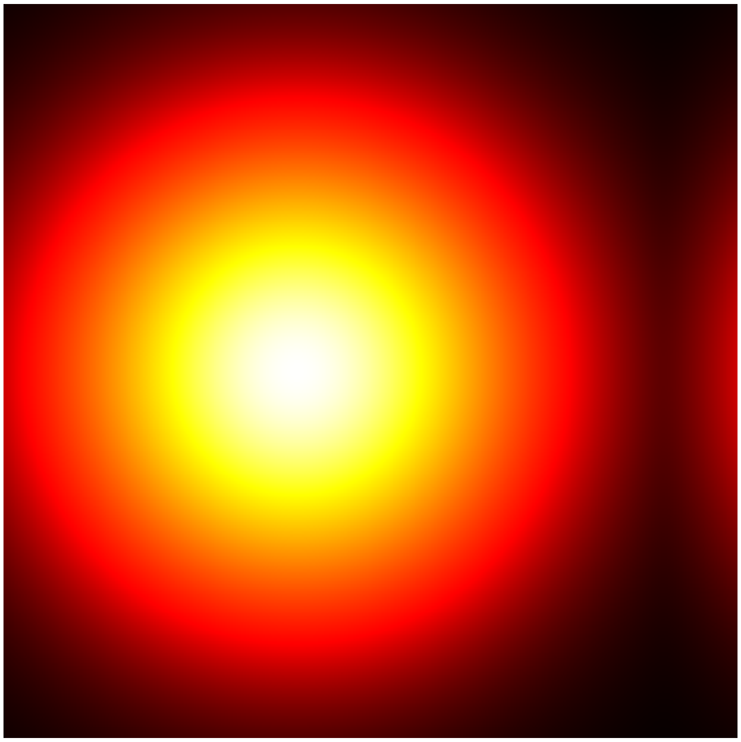

Figure AT. Final distribution after simulation using 2D Upwind advection scheme under periodic boundary conditions. The velocity field is uniform under x axis only. A whole period was almost simulated, transporting the distribution from left to right.

PopulationGrid Convergence Tests

To test that the behavior of the PopulationGrid approximates that of a partial differential equation when the population sizes are sufficiently large, and to ensure proper operation of the PopulationGrid and Multinomial Calculator, we tested the global L_1,L_2, and L_∞ convergence of the error between models with sizes 5, 15, 45, and 135 lattice points per spatial dimension. For the starting condition we used the probability density of a normal distribution with standard deviation 0.2 * dimension length, centered at the middle of the domain. This distribution was scaled such that the central position had probability 1, and the binomial was used to stochastically generate starting populations at each lattice position based on this probability distribution and a population size of 1e9.

We adjusted the time-step to compensate for the space step increments, simulating 10, 90, 810, and 7290 time-steps respectively. We used a constant "diffusion" rate of $0.01\frac{dx^2}{dt}$ and a zero flux boundary. Diffusion was modeled as Brownian motion of discrete agents, using the Multinomial Calculator to generate the numbers of agents that would move between lattice positions. The results for 1D, 2D, and 3D PopulationGrids are plotted below.

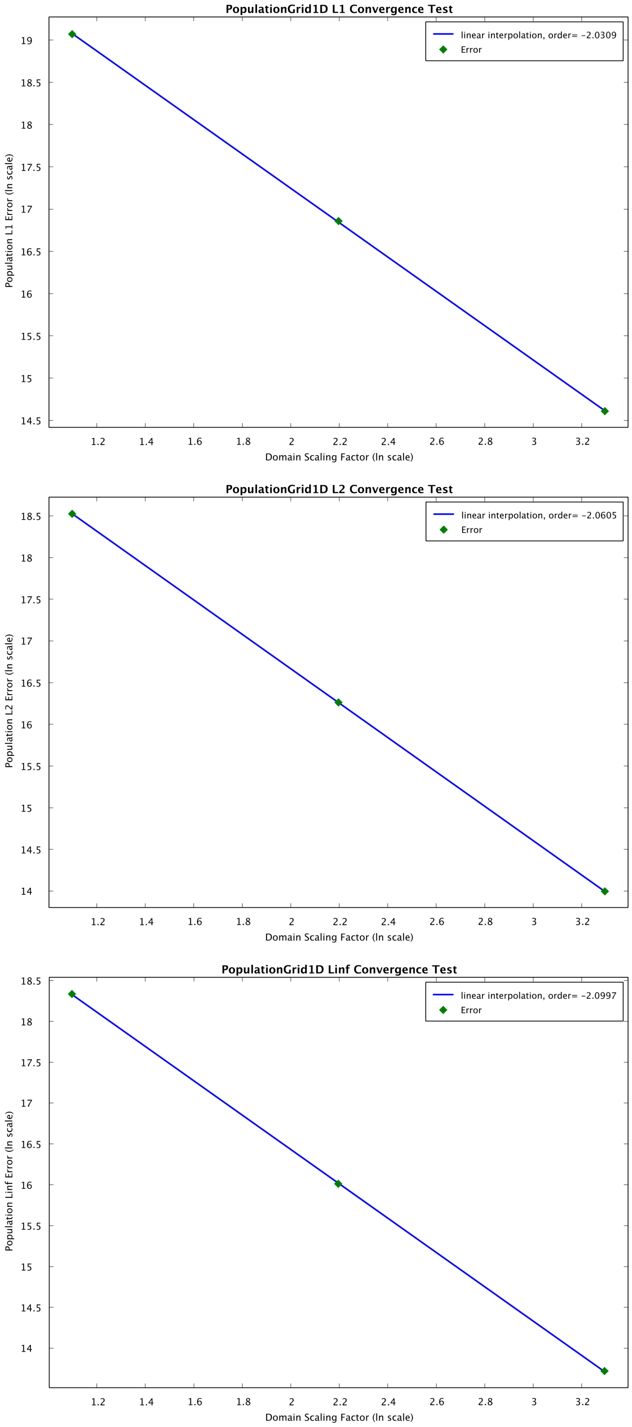

Figure AU. Space Convergence tests for the 1D Population Grid scheme with Von-Neumann boundary conditions and a centered gaussian initial condition.

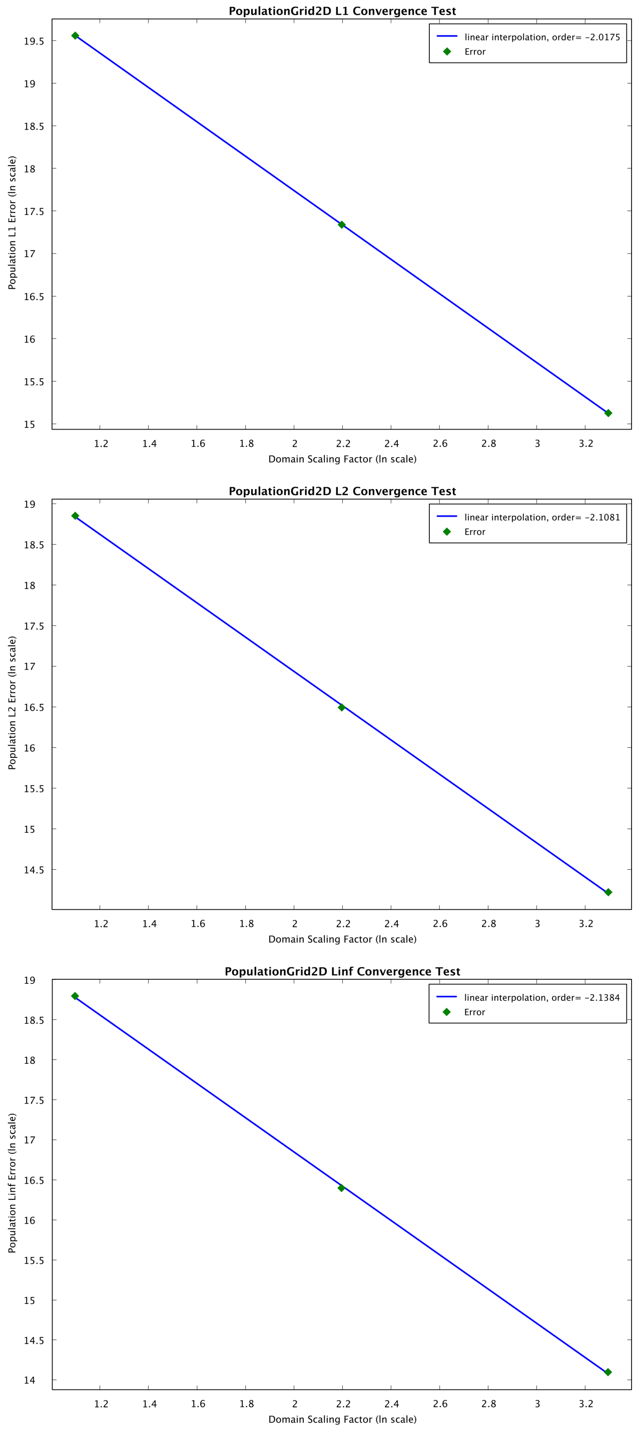

Figure AV. Space Convergence tests for the 2D Population Grid scheme with Von-Neumann boundary conditions and a centered gaussian initial condition.

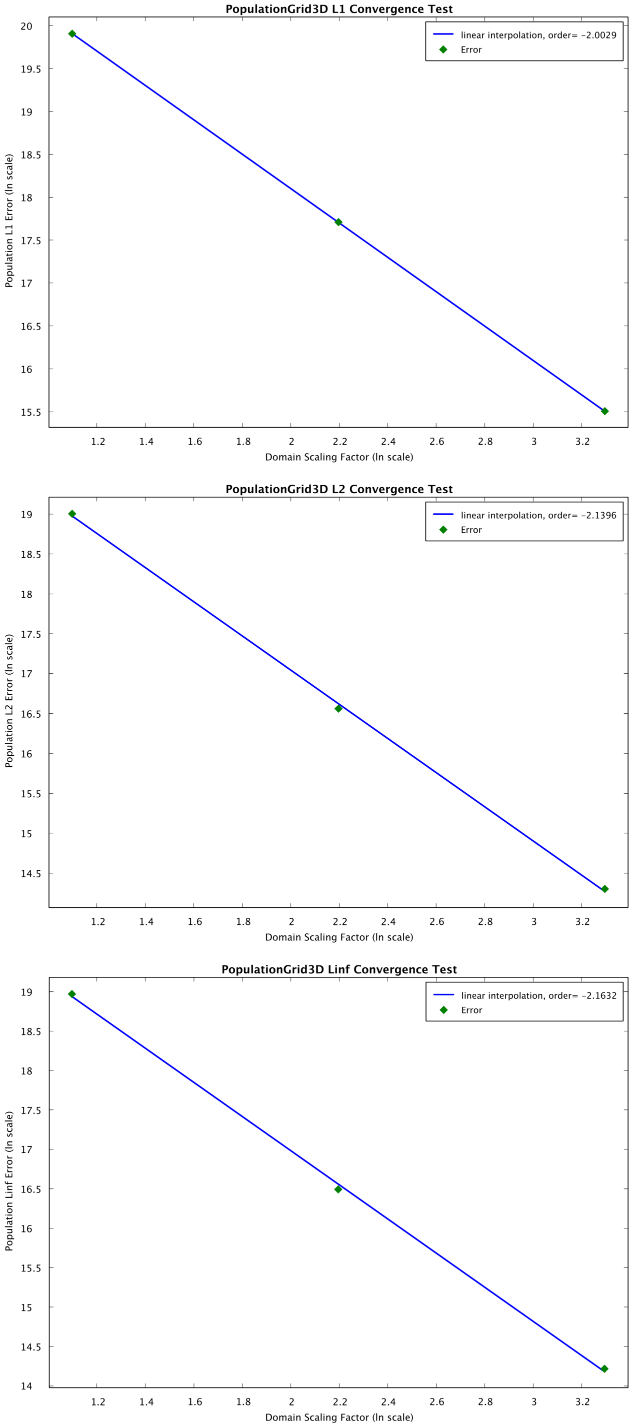

Figure AW. Space Convergence tests for the 3D Population Grid scheme with Von-Neumann boundary conditions and a centered gaussian initial condition.

Time Convergence Tests

PopulationGrid Convergence Tests

The PopulationGrid convergence tests assess the convergence of the PopulationGrid diffusion function. They are designed very consistently, with 10 lattice points per dimension for each model run, 100 timesteps, and consistent diffusion coefficient values of 0.01. The number of timesteps was scaled up consistently by a factor of 2 for the collection of each subsequent data point. The agents are initially arranged using a centered gaussian to calculate the probability of placement, with a maximum number of agents per lattice position of 2000000000 The results are shown below.

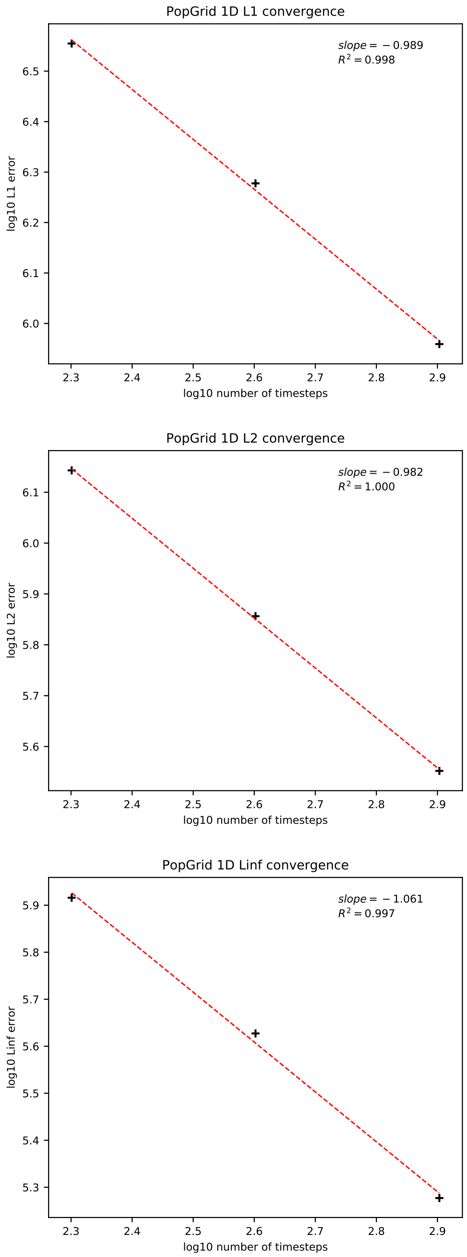

Figure AX. Time Convergence tests for the 1D Population Grid scheme with Von-Neumann boundary conditions and a centered gaussian initial condition.

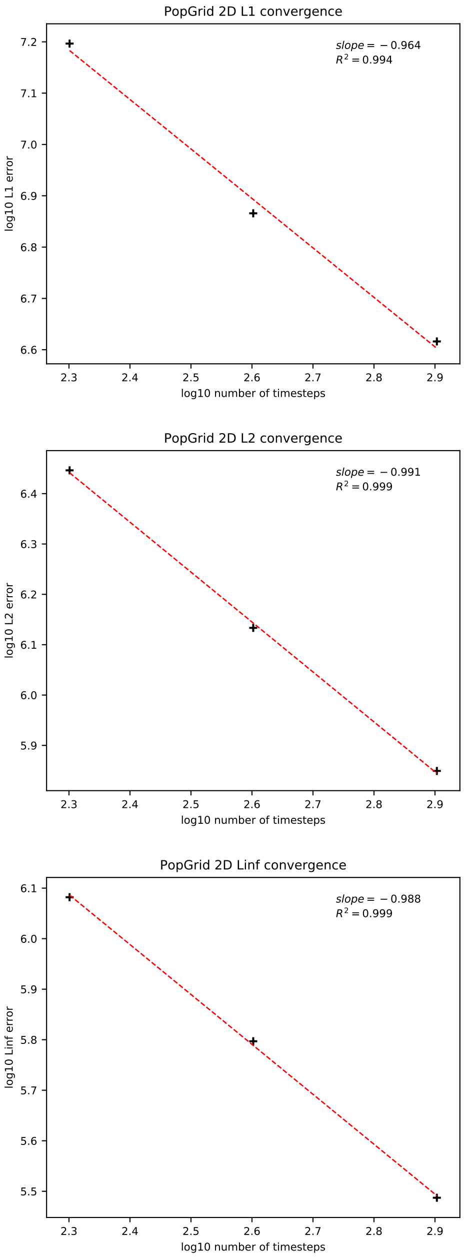

Figure AY. Time Convergence tests for the 2D Population Grid scheme with Von-Neumann boundary conditions and a centered gaussian initial condition.

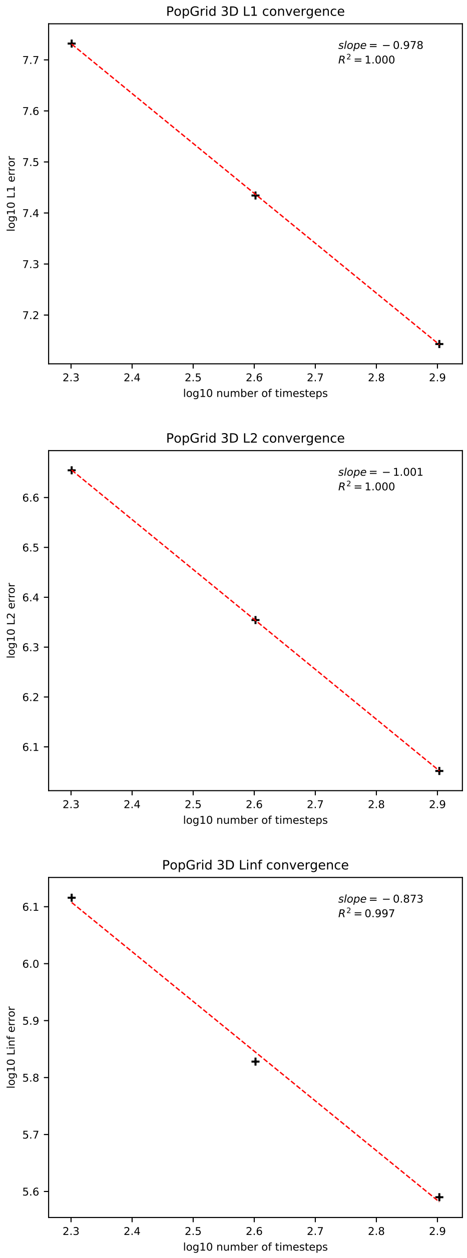

Figure AZ. Time Convergence tests for the 3D Population Grid scheme with Von-Neumann boundary conditions and a centered gaussian initial condition.

Unit Tests

One way in which we have ensured that HAL's algorithms perform properly is with unit testing. These tests are classified into 3 domains: Math Tests, PDE Tests, and Agent Tests. In all cases HAL's components have been corrected when necessary such that they pass all tests. Random number generations was done using a slightly modified

version of the Java 8 SplittableRandom class located in Tools/SplittableRN.

Math Tests

ProbScale Function

algorithm: For a probabilistic event with probability p per unit time, the probability that the event never occurs in some $dt \leq 1$ is $(1-p)^{dt}$ so the probability that the event occurs in timestep dt is $1-(1-p)^{dt}$.

arguments: p, dt

sets tried: All combinations of p, dt from $s_p = \{0.5, 0.01, 0.0001\}$ and $s_{dt} = \{1.0, 0.5, 0.25, 0.1, 0.01\}$

success criteria: Mean and Standard deviation of the successful outcomes of 1000 trials must be within $\frac{\sigma}{4}$ of predicted mean and standard deviation value, calculated by $\bar{x} = 1000p$ and $\sigma = \sqrt{1000p(1-p)}$. A trial is considered successful if over the duration of unit time, the probabilistic event occurs at least once.

Binomial Sampler

algorithm: Primarily uses code from the Colt Binomial class, and uses code from the numpy.random.binomial function when the Colt Binomial class was inaccurate.

arguments: n, p

sets tried: All combinations of n, p from $s_n = \{0.0001, 0.01, 0.5, 0.9, 0.9999\}$ and $s_p = \{1, 10, 1000, 1000000\}$

success criteria: Mean and Standard deviation of 100000 trials must be within $\frac{\sigma}{4}$ of predicted mean and standard deviation value, calculated by $\bar{x} = np$ and $\sigma = \sqrt{np(1-p)}$.

Multinomial Sampler

algorithm: the "conditional" method

arguments: n, ps

sets tried: 3 cases: $n = 1000, ps = \{0.1, 0.1, 0.1, 0.2\}$, $n = 10, ps = \{0.1, 0.1, 0.1\}$, and $n = 10, ps = \{0.01, 0.2, 0.5\}$

success criteria: means and standard deviations of n with each p_i must be within $\frac{\sigma}{4}$ of predicted mean and standard deviation value, calculated by $\bar{x} = np_i$ and $\sigma = \sqrt{np(1-p_i)}$.

Gaussian Sampler

algorithm: Ziggaraut Algorithm

arguments: mean, std

sets tried: All combinations of m and s from $s_m = (0, 100000, -100000)$ and $s_s = (0.0, 0.0001, 1, 100, 10000)$

success criteria: Mean and Standard deviation of 100000 trials must be within $\frac{\sigma}{4}$ of m and s arguments.

Runge Kutta 4

algorithm: 4th order Runge Kutta

arguments: DerivativeFunction, SolutionFunction, x_0, d_t t_f,

sets tried: DerivativeFunction:$\frac{dy}{dt} = y$, SolutionFunction:$y(t) = e^t$,$x_0 = 1$, $d_t = 0.01$ $t_f = 100$

DerivativeFunction:$\frac{dy}{dt} = y(1-y)$, SolutionFunction:$y(t) = \frac{e^t}{999+e^t}$, $x_0 = 0.001$, $d_t = 0.01$, $t_f = 100$

success criteria: Relative Error at all steps must be < 0.01

Runge Kutta Fehlberg 4,5

algorithm: Runge–Kutta–Fehlberg method 4(5)

arguments: DerivativeFunction, SolutionFunction, x_0, d_t t_f,

sets tried: DerivativeFunction:$\frac{dy}{dt} = y$, SolutionFunction:$y(t) = e^t$, $x_0 = 1$, $d_t = 0.01$ $t_f = 10$

DerivativeFunction:$\frac{dy}{dt} = y(1-y)$, SolutionFunction:$y(t) = \frac{e^t}{999+e^t}$, $x_0 = 0.001$, $d_t = 0.01$, $t_f = 10$

success criteria: Relative Error at all steps must be < 0.01

PDE Tests

1D Diffusion

algorithm:Forward difference in time and second order central difference in space (FTCS)

model: boundary conditions: $C(0) = 0, C(5) = 1$, domain: $\in [0, 5]$, diffusion constant $= 0.1$

exact solution: At steady state, $C(x) = x/5$

success criteria: after the model reaches steady state ($\|C^{n+1}_{\Delta x} - C^n_{\Delta x}\|_\infty < 0.0001$) the difference between the exact solution and the model lattice at all points must be less than 0.01 ($\|C - C^n_{\Delta x}\|_\infty < 0.01$)

2D Diffusion

algorithm:Forward difference in time and second order central difference in space (FTCS)

model: boundary conditions: $C(0, y) = 0, C(5, y) = 1$, domain: $\in x = [0, 5], y = [0, 6]$, diffusion constant $= 0.1$

exact solution: At steady state, $C(x, y) = x/5$

success criteria: after the model reaches steady state ($\|C^{n+1}_{\Delta x} - C^n_{\Delta x}\|_\infty < 0.0001$) the difference between the exact solution and the model lattice at all points must be less than 0.01 ($\|C - C^n_{\Delta x}\|_\infty < 0.01$)

3D Diffusion

algorithm:forward difference in time and second order central difference in space (FTCS)

model: boundary conditions: $C(0, y, z) = 0, C(5, y, z) = 1$, domain: $\in x = [0, 5], y = [0, 6], z = [0, 7]$, diffusion constant $= 0.1$

exact solution: At steady state, $C(x, y, z) = x/5$

success criteria: after the model reaches steady state ($\|C^{n+1}_{\Delta x} - C^n_{\Delta x}\|_\infty < 0.0001$) the difference between the exact solution and the model lattice at all points must be less than 0.01 ($\|C - C^n_{\Delta x}\|_\infty < 0.01$)

1D Advection

algorithm:First order upwind scheme

model: boundary conditions: $C(0) = 1$, domain: $\in [0, 5]$, advection velocity: $\Delta x = 0.1$

exact solution: At steady state, $C(x) = x/5$
success criteria: after the model reaches steady state ($\|C^{n+1}_{\Delta x} - C^{n}_{\Delta x}\|_\infty < 0.0001$) the difference between the exact solution and the model lattice at all points must be less than 0.01 ($\|C - C^{n}_{\Delta x}\|_\infty < 0.01$)

2D Advection

algorithm:First order upwind scheme
model: boundary conditions: $C(0, y) = 1$, domain: $\in x = [0, 5], y = [0, 6]$, advection velocity: $\Delta x = 0.1$
exact solution: At steady state, $C(x) = x/5$
success criteria: after the model reaches steady state ($\|C^{n+1}_{\Delta x} - C^{n}_{\Delta x}\|_\infty < 0.0001$) the difference between the exact solution and the model lattice at all points must be less than 0.01 ($\|C - C^{n}_{\Delta x}\|_\infty < 0.01$)

3D Advection

algorithm:First order upwind scheme
model: boundary conditions: $C(0, y, z) = 1$, domain: $\in x = [0, 5], y = [0, 6], z = [0, 7]$, advection velocity: $\Delta x = 0.1$
exact solution: At steady state, $C(x) = x/5$
success criteria: after the model reaches steady state ($\|C^{n+1}_{\Delta x} - C^{n}_{\Delta x}\|_\infty < 0.0001$) the difference between the exact solution and the model lattice at all points must be less than 0.01 ($\|C - C^{n}_{\Delta x}\|_\infty < 0.01$)

Agent Tests

1D Movement On Lattice No Wraparound

grid type: AgentGrid1D, no wraparound, 100 lattice
agent initial condition; 100 AgentSQ1D agents at position (0)
step function: all Agents move $\Delta x = 1$, stopping at the boundary (using the MoveSafeSQ function), shuffle agent iteration
step number: 1000
success criteria: there should be 100 agents at position (99), and no agents anywhere else

1D Movement On Lattice With Wraparound

grid type: AgentGrid1D, with wraparound, 100 lattice
agent initial condition:100 AgentSQ1D agents at position (0)
step function: all Agents move $\Delta x = 1$, wrapping around the boundary (using the MoveSafeSQ function), shuffle agent iteration.
step number: 1050
success criteria: there should be 100 agents at position (50), and no agents anywhere else

1D BirthDeath On Lattice

grid type: AgentGrid1D, no wraparound, 100 lattice
agent initial condition:100 AgentSQ1D agents at position (0)
step function: all Agents move, choosing randomly from $\Delta x = \{-1, 0, 1\}$. the first 50 agents to be iterated over are disposed, a new agent is created at the positions of the last 50, agent iteration is shuffled

step number: 100
success criteria: the total agent population on the grid should be 100

1D Movement Off Lattice No Wraparound

grid type: AgentGrid1D, no wraparound, 100 lattice
agent initial condition:100 AgentPT1D agents at position (0)
step function: all Agents move $\Delta x = 0.25$, stopping at the boundary (using the MoveSafePT function), shuffle agent iteration
step number: 1000
success criteria: there should be 100 agents at position (99), and no agents anywhere else

1D Movement Off Lattice With Wraparound

grid type: AgentGrid1D, with wraparound, 100 lattice
agent initial condition:100 AgentPT1D agents at position (0)
step function: all Agents move $\Delta x = 0.25$, wrapping around the boundary (using the MoveSafePT function), shuffle agent iteration
step number: 1000
success criteria: there should be 100 agents at position (50), and no agents anywhere else

1D BirthDeath Off Lattice

grid type: AgentGrid1D, no wraparound, 100 lattice
agent initial condition:100 AgentPT1D agents at position (0)
step function: all Agents move within the range $\Delta x = [-0.5, 0.5]$. The first 50 agents to be iterated over are disposed, a new agent is created at the positions of the last 50, agent iteration is shuffled
step number: 100
success criteria: the total agent population on the grid should be 100

2D Movement On Lattice No Wraparound

grid type: AgentGrid2D, no wraparound, 100x100 lattice
agent initial condition; 100 AgentSQ2D agents at position (0,0)
step function: all Agents move $\Delta x = 1, \Delta y = 1$, stopping at the boundary (using the MoveSafeSQ function), shuffle agent iteration
step number: 1000
success criteria: there should be 100 agents at position (99,99), and no agents anywhere else

2D Movement On Lattice With Wraparound

grid type: AgentGrid1D, with wraparound, 100x100 lattice
agent initial condition:100 AgentSQ2D agents at position (0,0)
step function: all Agents move $\Delta x = 1, \Delta y = 1$, wrapping around the boundary (using the MoveSafeSQ function), shuffle agent iteration
step number: 1050
success criteria: there should be 100 agents at position (50,50), and no agents anywhere else

2D BirthDeath On Lattice

grid type: AgentGrid2D, no wraparound, 100x100 lattice
agent initial condition:100 AgentSQ2D agents at position (0,0)
step function: all Agents move, choosing randomly from $\Delta x = \{-1, 0, 1\}, \Delta y = \{-1, 0, 1\}$. the first 50 agents to be iterated over are disposed, a new agent is created at the positions of the last 50, agent iteration is shuffled
step number: 100
success criteria: the total agent population on the grid should be 100

2D Movement Off Lattice No Wraparound

grid type: AgentGrid2D, no wraparound, 100x100 lattice
agent initial condition:100 AgentPT2D agents at position (0,0)
step function: all Agents move $\Delta x = 0.25, \Delta y = 0.25$, stopping at the boundary (using the MoveSafePT function), shuffle agent iteration
step number: 1000
success criteria: there should be 100 agents at position (99,99), and no agents anywhere else

2D Movement Off Lattice With Wraparound

grid type: AgentGrid2D, with wraparound, 100x100 lattice
agent initial condition:100 AgentPT2D agents at position (0,0)
step function: all Agents move $\Delta x = 0.25, \Delta y = 0.25$, wrapping around the boundary (using the MoveSafePT function), shuffle agent iteration
step number: 1000
success criteria: there should be 100 agents at position (50,50), and no agents anywhere else

2D BirthDeath Off Lattice

grid type: AgentGrid2D, no wraparound, 100x100 lattice
agent initial condition:100 AgentPT2D agents at position (0,0)
step function: all Agents move within the range $\Delta x = [-0.5, 0.5] \Delta y = [-0.5, 0.5]$. The first 50 agents to be iterated over are disposed, a new agent is created at the positions of the last 50, agent iteration is shuffled
step number: 100
success criteria: the total agent population on the grid should be 100

3D Movement On Lattice No Wraparound

grid type: AgentGrid3D, no wraparound, 100x100x100 lattice
agent initial condition; 100 AgentSQ3D agents at position (0,0,0)
step function: all Agents move $\Delta x = 1, \Delta y = 1, \Delta z = 1$, stopping at the boundary (using the MoveSafeSQ function), shuffle agent iteration
step number: 1000
success criteria: there should be 100 agents at position (99,99,99), and no agents anywhere else

3D Movement On Lattice With Wraparound

grid type: AgentGrid3D, with wraparound, 100x100x100 lattice
agent initial condition:100 AgentSQ3D agents at position (0,0,0)

step function: all Agents move $\Delta x = 1, \Delta y = 1, \Delta z = 1$, wrapping around the boundary (using the MoveSafeSQ function), shuffle agent iteration

step number: 1050

success criteria: there should be 100 agents at position (50,50,50), and no agents anywhere else

3D BirthDeath On Lattice

grid type: AgentGrid3D, no wraparound, 100x100x100 lattice

agent initial condition:100 AgentSQ2D agents at position (0,0)

step function: all Agents move, choosing randomly from $\Delta x = \{-1, 0, 1\}, \Delta y = \{-1, 0, 1\}, \Delta z = \{-1, 0, 1\}$. the first 50 agents to be iterated over are disposed, a new agent is created at the positions of the last 50, agent iteration is shuffled

step number: 100

success criteria: the total agent population on the grid should be 100

3D Movement Off Lattice No Wraparound

grid type: AgentGrid3D, no wraparound, 100x100x100 lattice

agent initial condition:100 AgentPT2D agents at position (0,0,0)

step function: all Agents move $\Delta x = 0.25, \Delta y = 0.25, \Delta z = 0.25$, stopping at the boundary (using the MoveSafePT function), shuffle agent iteration

step number: 1000

success criteria: there should be 100 agents at position (99,99), and no agents anywhere else

3D Movement Off Lattice With Wraparound

grid type: AgentGrid3D, with wraparound, 100x100x100 lattice

agent initial condition:100 AgentPT2D agents at position (0,0,0)

step function: all Agents move $\Delta x = 0.25, \Delta y = 0.25, \Delta z = 0.25$, wrapping around the boundary (using the MoveSafePT function), shuffle agent iteration

step number: 1000

success criteria: there should be 100 agents at position (50,50,50), and no agents anywhere else

3D BirthDeath Off Lattice

grid type: AgentGrid3D, no wraparound, 100x100x100 lattice

agent initial condition:100 AgentPT1D agents at position (0,0,0)

step function: all Agents move within the range $\Delta x = [-0.5, 0.5], \Delta y = [-0.5, 0.5], \Delta z = [-0.5, 0.5]$. The first 50 agents to be iterated over are disposed, a new agent is created at the positions of the last 50, agent iteration is shuffled

step number: 100

success criteria: the total agent population on the grid should be 100

PopulationGrid Tests

1D PopulationGrid Diffusion

grid type: PopulationGrid1D, 100 lattice, PDEGrid1D, 100 lattice, zero flux boundary

initial condition:10000 population position (50), 0 elsewhere, PDE 10000 concentration at position (50), 0 elsewhere

step function: population diffusion with movement probability 0.1 via multinomial probability sampling, PDE diffusion with rate constant 0.1 using FCTS

step number: 1000

success criteria: The difference between the population number at each lattice position and the PDE concentration at each lattice position should be less than 100

2D PopulationGrid Diffusion

grid type: PopulationGrid2D, 20x20 lattice, PDEGrid2D, 20x20 lattice, zero flux boundary

initial condition:10000 population position (10,10), 0 elsewhere, PDE 10000 concentration at position (10,10), 0 elsewhere

step function: population diffusion with movement probability 0.1 via multinomial probability sampling, PDE diffusion with rate constant 0.1 using FCTS

step number: 200

success criteria: The difference between the population number at each lattice position and the PDE concentration at each lattice position should be less than 100

3D PopulationGrid Diffusion

grid type: PopulationGrid3D, 10x10x10 lattice, PDEGrid3D, 10x10x10 lattice, zero flux boundary

initial condition:10000 population position (10), 0 elsewhere, PDE 10000 concentration at position (10), 0 elsewhere

step function: population diffusion with movement probability 0.1 via multinomial probability sampling, PDE diffusion with rate constant 0.1 using FCTS

step number: 100

success criteria: The difference between the population number at each lattice position and the PDE concentration at each lattice position should be less than 100
